# Elucidating the Chirality-Induced Spin Selectivity Effect of Co-Doped NiO Deposited on Ni Foam for Highly Stable Zn–Air Batteries

**DOI:** 10.1021/acsami.4c20630

**Published:** 2025-03-13

**Authors:** Young Sun Park, Jeongyoub Lee, Hyungsoo Lee, Jung Been Park, Juwon Yun, Chan Uk Lee, Subin Moon, Soobin Lee, Sumin Kim, Jun Hwan Kim, Donghyun Kim, Jimin Han, Dong-Wan Kim, Jooho Moon

**Affiliations:** † Department of Materials Science and Engineering, Yonsei University, Seoul 03722, Republic of Korea; ‡ School of Civil, Environmental, and Architectural Engineering, Korea University, Seoul 02841, Republic of Korea

**Keywords:** zinc−air battery, oxygen evolution reaction, chirality-induced spin selectivity, spin polarization, heteroatom-doped oxygen catalyst

## Abstract

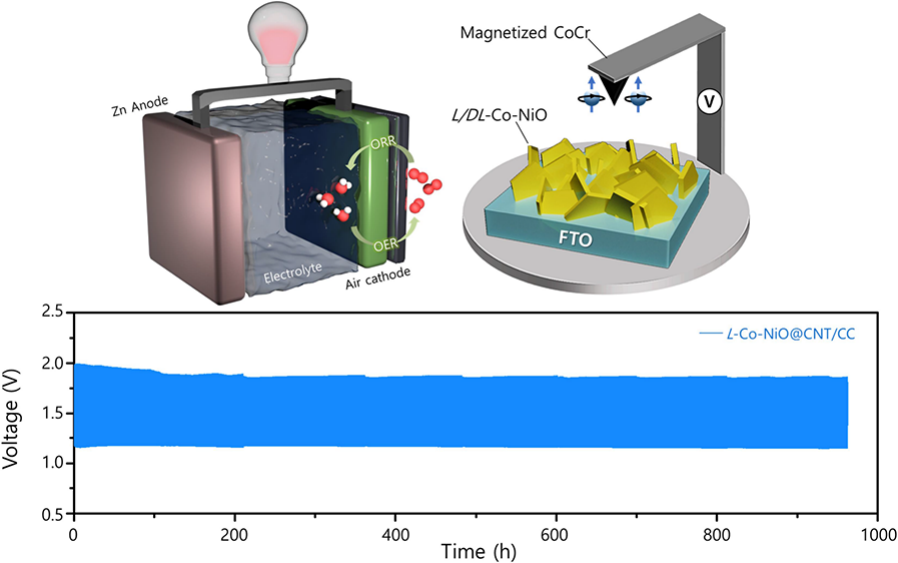

The urgent need to alleviate global warming and limit the consumption of fossil fuels has prompted the development of rechargeable Zn–air batteries (ZABs) considering their superior energy density, safety, and cost-effectiveness. However, the sluggish reaction kinetics of the oxygen evolution reaction (OER) and the unfavorable properties of conventional OER catalysts (including low electrical conductivity and the use of active site-blocking binders) hinder the development of practically viable ZABs. Herein, we report a distinct approach for directly synthesizing cobalt-doped nickel oxide (Co-NiO) with a chiral structure on porous Ni foam via a one-step hydrothermal process. The chirality-induced spin selectivity (CISS) boosts the OER kinetics, while Co doping elevates the electrical conductivity and the abundance of active sites on the catalyst. The chiral Co-NiO demonstrates an OER current density of 10 mA cm^–2^ at 1.58 V versus the reversible hydrogen electrode, outperforming both achiral Co-NiO and undoped NiO. Furthermore, a chiral Co-NiO-based rechargeable ZAB demonstrates a high open-circuit potential (1.57 V), a low charge/discharge overpotential (0.71 V), and excellent stability for 960 h (40 days) because the CISS effect mitigates the production of the corrosive singlet oxygen. These results represent a prominent pathway for the advancement of ZABs using the low-cost oxygen evolution catalyst modulated by the CISS effect and heteroatomic doping.

## Introduction

To alleviate the increasingly severe greenhouse effect and the continued depletion of conventional fossil fuels, researchers focus on the development of renewable energy storage and conversion systems.^1,2^ Recently, metal–air (e.g., Li–air, Na–air, Zn–air, etc.) batteries have been extensively studied as a viable future energy storage system due to their decent energy density and use of earth-abundant air.^3−6^ Especially, rechargeable Zn–air batteries (ZABs) have attracted considerable attention as the most promising candidate for next-generation energy storage devices owing to their high theoretical energy density (1086 W h kg^–1^), safe utilization of aqueous electrolytes, as well as nonvolatile nature and cost-effectiveness of Zn resources.^7−9^ However, the sluggish kinetics of the oxygen evolution reaction (OER)—the anodic reaction in a ZAB—represent a major bottleneck in improving the energy efficiency of ZABs.^10,11^ While compounds based on novel metals (e.g., ruthenium (Ru) and iridium (Ir)) have served as effective OER catalysts, the high price of such novel metals and their performance degradation, which occurs during consecutive OER because of the electrochemical dissolution of active sites, hinder the practical development of high-performance ZABs.^12,13^ Therefore, to develop viable rechargeable ZABs, it is necessary to identify an efficient earth-abundant catalyst that can not only enhance the OER performance but also exhibit superior stability under extremely oxidative conditions.

The OER activity of electrocatalysts is primarily affected by the abundance of their surface active sites and their electrical conductivity.^14,15^ In this regard, to maximize the abundance of the active sites on catalysts, various strategies have recently been developed to synthesize catalysts by directly depositing them on highly porous foam-type current collectors (i.e., Ni foam, graphene foam, and carbon cloth) without using a binder.^16,17^ Heteroatomic doping is another effective approach for enhancing the electrical conductivity of catalysts and inducing lattice distortion, which is beneficial for increasing electrocatalytic activity.^18^ For example, Sanchez et al. directly deposited nickel–cobalt–manganese sulfide (NiCoMnS_*x*_) nanosheets on a porous graphene foam via electrochemical deposition and utilized the fabricated material as an air cathode for ZABs.^19^ This electrode exhibited an anodic potential of 1.59 V versus a reversible hydrogen electrode (V_RHE_) at a current density of 10 mA cm^–2^, which is lower than those of commercial Ru-based catalysts (1.63 V_RHE_). Furthermore, Behera et al. demonstrated that copper (Cu) doping can increase not only the electrical conductivity of cobalt oxide (Co_3_O_4_)-based electrocatalysts but also the amount of oxygen vacancies on the catalyst surface, which serve as active sites for the OER.^20^ The Cu-doped Co_3_O_4_ catalyst exhibited a lower oxidation potential (1.59 V_RHE_) compared with the undoped Co_3_O_4_ (1.65 V_RHE_) at a current density of 10 mA cm^–2^. Likewise, extensive research has been devoted to rationally designing effective OER catalysts for the implementation of high-performance ZABs. However, lowering the overpotential of the OER to the theoretical level remains exceedingly challenging.

The OER is a multistep and multielectron reaction, which involves several spin-dependent electrochemical reactions based on the quantum mechanical perspective.^21^ Specifically, the formation of spin-aligned intermediate radicals (^·^OH) is required for the prompt generation of triplet oxygen molecules (^3^O_2_) in a ^3^Σ_g_^–^ triplet ground state.^22^ If the spins of ^·^OH are not aligned, energetically unfavorable singlet oxygen molecules (^1^O_2_) and even byproducts such as hydrogen peroxide (H_2_O_2_) are produced.^23^ Since ^1^O_2_ is in a higher-energy state (^1^Δ_g_ singlet excited state) and is more chemically reactive than ^3^O_2_, the singlet-mediated processes significantly increase the OER overpotential and exacerbate electrode corrosion during the operation of ZABs.^24−26^ Therefore, based on the principles of quantum theory, the spin orientation of the electrons evolved during the OER should be regulated to energetically and entropically accelerate the formation of ^3^O_2_, thereby minimizing the OER overpotential and the performance degradation of ZABs. Chirality-induced spin selectivity (CISS), a recently introduced strategy, enables efficient control over the spin of electrons transported through electrocatalysts.^25,27,28^ The CISS effect involves the chiral structure acting as a spin polarizer that can align the spin states of electrons as they migrate through the chiral structure.^29,30^ The CISS effect can be easily induced by imparting structural chirality to various electrocatalysts through the integration of chiral organic molecules with catalytic materials.^31^ Indeed, several metal oxides (NiO_*x*_, CuO, and Fe_3_O_4_) synthesized in the presence of chiral organic molecules exhibit distinct structural chirality, which suggests that the resulting CISS effect could enhance the production of energetically favorable ^3^O_2_.^32−34^ However, the previously reported oxide catalysts were characterized by low electrical conductivity or obtained in powder form, which needed to be deposited on a substrate by using additional binder materials, resulting in a substantial decrease in active sites.

In this study, we successfully fabricated cobalt-doped nickel oxide (Co-NiO) with a chiral structure as an optimized oxidative oxygen-ergomer (OER) electrocatalyst for ZABs. By combining the advantages of the CISS effect and heteroatomic doping, the chiral Co-NiO accelerates the OER kinetics via spin-modulated electrochemistry, in addition to exhibiting enhanced hole conductivity and a larger amount of active sites. Moreover, as chiral Co-NiO can be directly synthesized on a porous Ni foam (NF) via a one-step hydrothermal process, the amount of active sites can be maximized. The prepared chiral Co-NiO displayed effective spin selectivity, achieving 60% spin polarization as evidenced by magnetic conductive-probe atomic microscopy. The chiral Co-NiO-based OER catalyst delivered a current density of 10 mA cm^–2^ at an anodic potential of 1.58 V_RHE_, which is 46 and 41 mV less than those of its achiral Co-NiO and undoped chiral NiO counterparts, respectively. Additionally, in-depth electrochemical characterizations explicitly verified that the synergistic behaviors of the CISS and doping effects produced a significant increase in OER activity. Moreover, when chiral Co-NiO was utilized as the catalyst for the air cathode, the rechargeable ZAB achieved a high open-circuit potential (1.57 V), a low voltage gap (0.71 V), and stable operation for 960 h (40 days) due to the suppressed production of ^1^O_2_. Our results suggest that the chiral OER catalyst meditated by CISS and doping effects can be a breakthrough for high-performance stable ZABs.

## Results and Discussion

**Figure 1:**
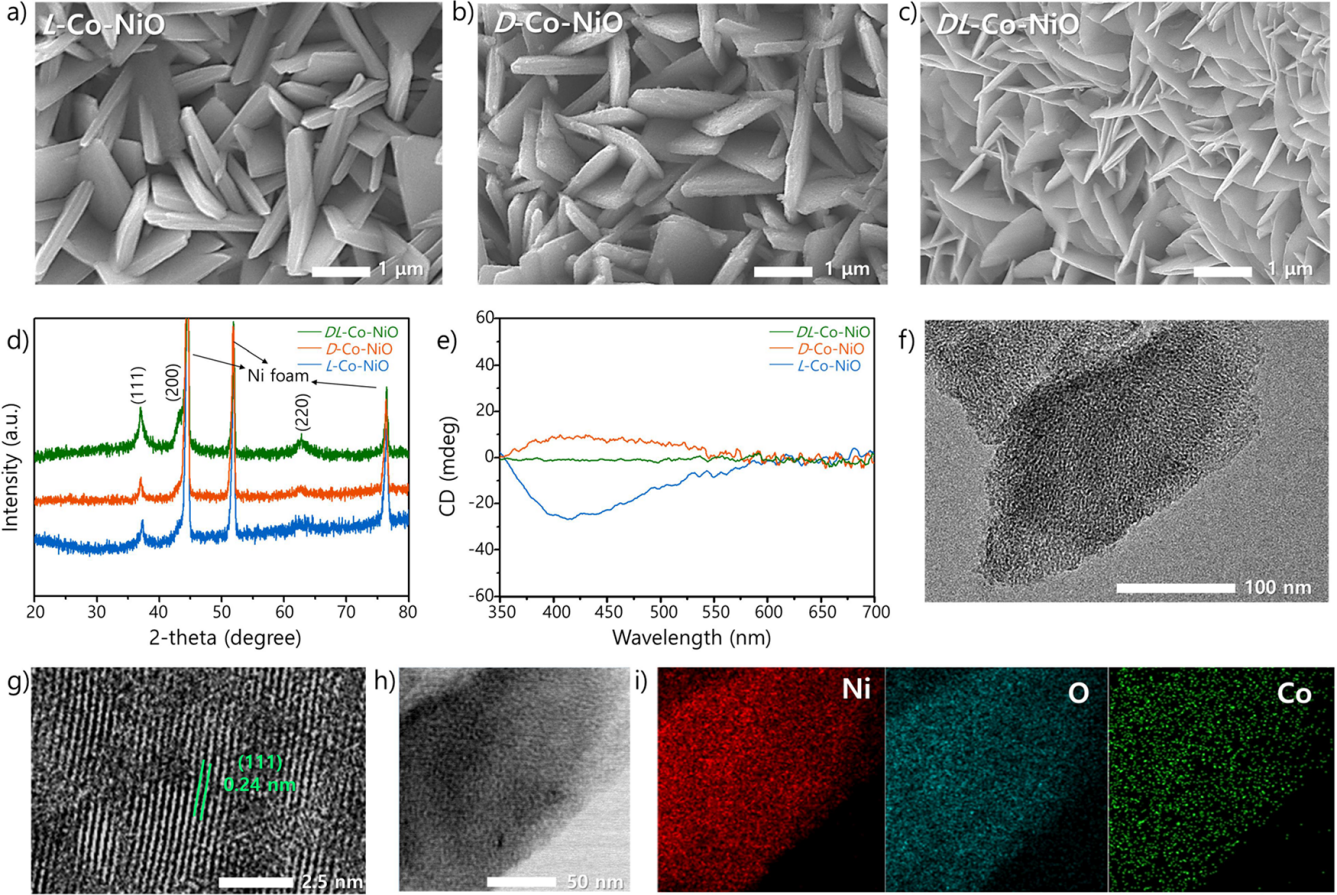
SEM images of (a) *L*-Co-NiO, (b) *D*-Co-NiO, and (c) *DL*-Co-NiO on the NF. (d) XRD patterns of *L*/*D*/*DL*-Co-NiO on the NF. (e) CD spectra of *L*/*D*/*DL*-Co-NiO on FTO, (f) TEM image, (g) HR-TEM image, (h) STEM image, and (i) EDX mapping of the *L*-Co-NiO nanoplate.

The chiral-structured Co-NiO was decorated on a mesoporous NF current collector through hydrothermal synthesis, followed by a calcination process. The synthesis solution comprised NiCl_2_·6H_2_O, proline, and ammonia, along with CoCl_2_·6H_2_O with a molar concentration of 50% relative to NiCl_2_·6H_2_O for heteroatomic doping (see the Experimental Section for details). *L*-proline and *D*-proline were employed as chiral inducers to synthesize chiral-mesostructured Co-NiO, and the resulting electrodes were denoted as *L*-Co-NiO and *D*-Co-NiO, respectively. Moreover, racemic *DL*-proline was utilized to prepare the achiral *DL*-Co-NiO counterpart. Subsequently, the morphologies of the three samples were examined via scanning electron microscopy (SEM), which demonstrated nanoplate arrays deposited on the NF in all samples (Figure 1a–c). Despite the similar nanoplate morphologies of the three catalysts, high-magnification SEM analysis revealed differences in the thickness and distortion of the nanoplates due to the use of chiral organic ligands. As shown in Figure S1a,b, the *L*-Co-NiO and *D*-Co-NiO exhibited thick nanoplate-based microstructures with pronounced distortion. This distortion can be attributed to the asymmetric coordination of *L*/*D*-proline with Ni and Co ions. Specifically, a peptide bond between the amino groups and the carboxyl groups of *L*/*D*-proline promotes the helical arrangement of these organic molecules.^35^ During the hydrothermal process, the amino groups chelate with the Ni and/or Co ions and induce a symmetry-breaking effect; this results in the formation of chiral-mesostructured NiO with distorted nanoplates, as verified in previous reports.^30,36^ In contrast, *DL*-Co-NiO exhibited a thin nanoplate-based structure without any distortion (i.e., an achiral microstructure; Figure S1c). Specifically, the average nanoplate thickness was measured to be approximately 282, 246, and 153 nm for *L*-Co-NiO, *D*-Co-NiO, and *DL*-Co-NiO, respectively. The larger size of the *L*/*D*-Co-NiO nanoplates compared with that of the *DL*-Co-NiO nanoplates can be ascribed to the fact that the chiral arrangement of the *L*/*D*-proline during hydrothermal synthesis led to the growth of distorted nanocrystals; this not only reduced the nucleation rate but also limited the number of nuclei from which large nanoplates with low density were obtainable.^30,37,38^

To obtain the crystallographic information on the three synthesized catalysts, X-ray diffraction (XRD) analysis was performed (Figure 1d). All three samples displayed diffraction peaks at 37°, 43.46°, and 63.56°, which correspond to the (111), (200), and (220) crystal planes of NiO, respectively.^39^ This observation implies that Co-NiO exhibited phase purity without any secondary phases and/or impurities. Despite the nanoplates of *L*/*D*-Co-NiO being larger than those of *DL*-Co-NiO, the crystallinity of *L*/*D*-Co-NiO was lower than that of *DL*-Co-NiO due to the structural deformation caused by the chiral ligand–induced distorted nanocrystal growth. Furthermore, to investigate the effect of Co doping on the structural properties of NiO, SEM and XRD measurements were also conducted on *L*-NiO, which was prepared using the same hydrothermal procedure as that for *L*-Co-NiO, only without the addition of CoCl_2_·6H_2_O (Figure S2). The nanoplate-based microstructure of *L*-NiO was nearly identical to the morphology of *L*-Co-NiO (Figure S2a). In contrast, the intensities of the XRD diffraction peaks of *L*-NiO were higher than those of *L*-Co-NiO; moreover, the NiO(111) peak of *L*-NiO was located at a lower angle than that of *L*-Co-NiO due to the atomic size of Co^2+^ being smaller than that of Ni^2+^ (Figure S2b).^40,41^ This result indicates that Co doping induced a lattice distortion in the NiO-based nanoplates, enabling the generation of abundant active sites for electrochemical reactions.

To confirm the chirality of *L*/*D*/*DL*-Co-NiO, we examined the chiroptical properties of the three catalysts through transmission circular dichroism (CD) spectroscopy. The three NiO-based catalysts were deposited on fluorine-doped tin oxide (FTO) via the same hydrothermal process to determine their transmittance. As seen in Figure 1e, the CD spectra of *L*/*D*-Co-NiO demonstrated signals with opposite directions due to the different handedness of the *L*/*D*-proline, while the spectrum for *DL*-Co-NiO exhibited no CD signal. The CD signal in the spectra of the *L*/*D*-Co-NiO samples was detected at wavelengths shorter than 600 nm, corresponding to the edge of light absorption (Figure S3), which is consistent with a previous report.^30^ The higher intensity of the *L*-Co-NiO CD signals relative to that of the *D*-Co-NiO signals can be attributed to the difference in purity between *L*-proline (99.5%) and *D*-proline (99%), which promotes the growth of a chiral-based nanostructure.^42^ Moreover, to investigate whether the CD signals originated from remanent chiral organic molecules at the surface of Co-NiO, Raman analysis was performed on the three catalysts decorated on FTO. The Raman spectra of all three samples exhibited only vibrational modes of phase-pure NiO at around 532 and 1065 cm^–1^, without any characteristic peaks related to proline (Figure S4).^43^ This verifies that the chirality of the chiral organic ligand was successfully transferred to Co-NiO nanocrystals during the hydrothermal synthesis.

To elucidate the crystalline structure and elemental distribution of *L*-Co-NiO, the nanoplate was detached from the NF via ultrasonication before being investigated by transmission electron microscopy (TEM). The low-magnification TEM image of *L*-Co-NiO (Figure 1f) displays the nanoplate morphology confirmed in the SEM image (Figure 1a). A high-resolution TEM (HR-TEM) image of *L*-Co-NiO was also obtained, revealing a lattice distance of 0.24 nm; this is consistent with the interplanar spacing of the (111) plane of NiO (Figure 1g),^44^ which corroborates the XRD measurement. The HR-TEM images and three consecutive fast Fourier transform (FFT) images of the *L*-Co-NiO nanoplate were investigated further. Remarkably, Figure S5 illustrates that the FFT patterns of three different nanoplates exhibited anticlockwise rotations from the left (A) point to the right (C) point, highlighting that *L*-Co-NiO was assembled into helically distorted nanoplates.^36^ In contrast, the nanoplates of *D*-Co-NiO exhibited clockwise rotations of FFT patterns because of the opposite handedness of *L*/*D*-Co-NiO (Figure S6). Moreover, the degree of rotation in the atomic arrangement of nanoplates in *D*-Co-NiO was lower than that of *L*-Co-NiO, and one of the nanoplates displayed the absence of rotation, which is well matched with the results of CD measurement. Meanwhile, the rotation of FFT patterns in three different nanoplates of *DL*-Co-NiO was not detected, indicative of the absence of chirality (Figure S7). Furthermore, the images obtained through scanning transmission electron microscopy (STEM) and energy-dispersive X-ray spectroscopy (EDX) mapping revealed a uniform distribution of Ni, O, and Co atoms in the *L*-Co-NiO nanoplate, confirming that the nanoplates had been successfully doped with Co (Figure 1h,i). The STEM and EDX analyses of *D*/*DL*-Co-NiO were also carried out (Figure S8), and the atomic ratios of Co to Ni in *L*/*D*/*DL*-Co-NiO were 4.15, 3.61, and 4.48%, respectively (Tables S1–S3). Thus, the difference in concentrations of Co in the three different samples was negligibly less than 1%.

**Figure 2:**
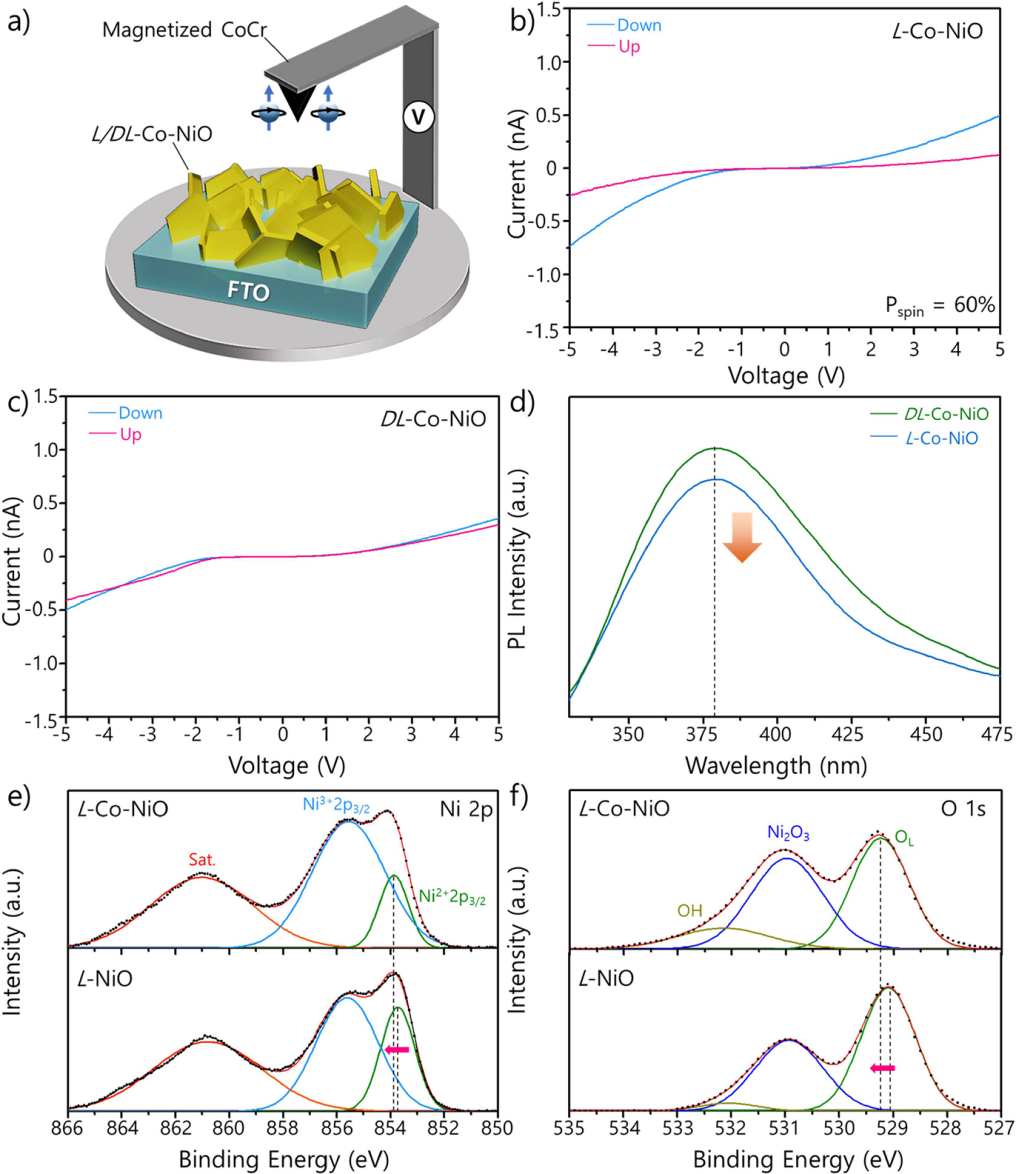
(a) Schematic illustration of mCP-AFM analysis. Average *I–V* curves for (b) *L*-Co-NiO and (c) *DL*-Co-NiO on FTO, estimated through mCP-AFM with a premagnetized tip along the upward or downward magnetic field orientation. (d) PL spectra of *L*/*DL*-Co-NiO on FTO. High-resolution XPS spectra of *L*-Co-NiO and *L*-NiO on the NF: deconvolution of (e) Ni 2p and (f) O 1s regions.

To examine the actual spin polarization produced by the CISS phenomenon in the *L*/*DL*-Co-NiO catalysts, the spin-selective charge transport was evaluated via magnetic conductive-probe atomic force microscopy (mCP-AFM), as illustrated in Figure 2a. The two catalysts were deposited on FTO substrates, as the roughness of NF substrates could be challenging for the AFM analysis. The Co-Cr-coated tip was premagnetized using a permanent magnet oriented in different directions (north and south) with respect to the as-prepared Co-NiO-based catalysts, and a bias was applied between the premagnetized tip and the substrate. To ensure reliable results, the *I–V* curves were measured at least 90 times from different positions on the two samples via contact-mode AFM. The average *I–V* curves for *L*/*DL*-Co-NiO are presented in Figure 2b,c, while Figure S9 shows the corresponding raw data. *L*-Co-NiO delivered notably different average current values for the two opposite magnetization orientations (Figure 2b). The current levels for downward magnetization were much higher than those for upward magnetization in the bias range of −5 to 5 V, while no substantial deviation in the current levels between the two orientations was observed for *DL*-Co-NiO (Figure 2c). The results of the mCP-AFM analysis demonstrate that a remarkable spin-relative current originated from the CISS effect in *L*-Co-NiO. The spin-polarization degree (*P*_spin_) can be utilized to estimate the anisotropy of spin-polarized current, as expressed in the following equation:
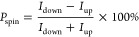
1where *I*_down_ and *I*_up_ are the evaluated current levels at a specific potential when the tip is magnetized in the downward and upward directions, respectively. The *P*_spin_ of *L*-Co-NiO was determined to be around 60% at 5 V. This value is larger than the values of chiral monolayer-based systems reported in previous studies, owing to the large scale of the chiral microstructure induced by the helically distorted nanocrystals of *L*-Co-NiO.^45−47^ Additionally, the *I–V* curves for *D*-Co-NiO obtained via mCP-AFM indicate that the current values for the upward orientation of magnetization were higher than those for the downward orientation (Figure S10). The *P*_spin_ of *D*-Co-NiO at 5 V was calculated to be 22%, which is lower than that of *L*-Co-NiO; this is explained by the different degrees of growth of the chiral-based nanostructure, as confirmed by the CD spectroscopy results (Figure 1e). Based on spin-dependent electrochemistry, the high spin-polarization capability of *L*-Co-NiO is likely to improve the OER performance with respect to the achiral counterpart.

Furthermore, steady-state photoluminescence (PL) spectroscopy was performed on *L*-Co-NiO and *DL*-Co-NiO loaded onto FTO substrates to observe the behaviors of the photoinduced charge carriers within the chiral and achiral structures, respectively (Figure 2d). The PL peaks of the two samples were observed at 380 nm, which is ascribed to radiative recombination, as reported recently.^48^ This result indicated that the band gaps of *L*/*DL*-Co-NiO were 3.26 eV. Interestingly, the PL emission peak representing the radiative recombination of *L*-Co-NiO exhibited a lower intensity than the corresponding peak for *DL*-Co-NiO did. Afterward, the band structures of *L*/*DL*-Co-NiO were determined using ultraviolet photoelectron spectroscopy (UPS) analysis because the behavior of photoinduced charge carriers depends on the band alignment between *L*/*DL*-Co-NiO and FTO (Figure S11a,b). Subsequently, the Fermi levels (*E*_f_) and valence bands (*E*_V_) of the two samples were calculated using the following equations:
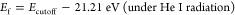
2
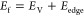
3

The results of the UPS analysis indicated that *L*/*DL*-Co-NiO revealed nearly identical secondary-electron cutoff (*E*_cutoff_) values of 16.41 and 16.42 eV, corresponding to *E*_f_ values of −4.79 and −4.80 eV, respectively. Moreover, the two different materials exhibited valence band edge (*E*_edge_) comparable values of 0.76 and 0.77 eV, indicating the values of *E*_V_ for *L*/*DL*-Co-NiO (−5.56 eV). Meanwhile, the conduction bands (*E*_C_) of *L*/*DL*-Co-NiO were −2.3 eV given the band gaps of *L*/*DL*-Co-NiO (3.26 eV). Therefore, the band structures of *L*/*DL*-Co-NiO were nearly identical, as illustrated in Figure S11c. It is well-known that the *E*_f_ of FTO is approximately −4.4 eV, which is higher than those of *L*/*DL*-Co-NiO, as demonstrated by several previous studies.^49,50^ When the *E*_f_ of a conductor is higher than that of a p-type semiconductor, the p-type Schottky contact can be formed, as seen in Figure S11d.^51^ Therefore, this band alignment between *L*/*DL*-Co-NiO and FTO allows photoinduced electrons to be transferred from *L*/*DL*-Co-NiO to FTO, while the photogenerated holes remain confined to *L*/*DL*-Co-NiO (Figure S11e).^52,53^ The CISS effect induced by this migration of photoexcited electrons from *L*-Co-NiO to FTO resulted in the reduced radiative recombination of *L*-Co-NiO compared to *DL*-Co-NiO.^22,54^ This is because the spin polarization resulting from the CISS effect suppresses the radiative recombination of photogenerated charge carriers.^22,54^ More specifically, when the photogenerated electrons of *L*/*DL*-Co-NiO are excited from the valence band to the conduction band, the spin state of the photoinduced holes in the valence band adopts the direction opposite to that of the electrons (Figure S12). Thereafter, because the work function of NiO exceeds that of FTO, the Schottky junction between the p-type NiO and FTO allows electrons to transfer from *L*/*DL*-Co-NiO to FTO, while the holes remain confined to *L*/*DL*-Co-NiO.^52,53^ As *DL*-Co-NiO lacks spin-alignment capability, its photoinduced electrons and holes can exhibit randomly aligned spin states, leading to spontaneous radiative recombination during the transport of electrons from *DL*/Co-NiO to FTO (Figure S12a). However, when photoinduced electrons are generated in *L*-Co-NiO, their spin states can be aligned in a specific direction during their transfer from *L*-Co-NiO to FTO (Figure S12b). Therefore, the spin-up and spin-down configurations of the electrons and holes in *L*-Co-NiO can have different probabilities, resulting in the suppression of radiative recombination based on the Pauli exclusion principle.^54^ This result corroborates the occurrence of spin polarization in *L*-Co-NiO due to the CISS effect. Furthermore, photoadsorption could be another reason for reduced radiative recombination. Therefore, after 1 sun illumination (AM 1.5G light) was irradiated on *L*/*DL*-Co-NiO on FTO for 10 min, the Raman measurements were immediately conducted to investigate the adsorbates induced by the photoadsorption of Co-NiO-based catalysts. As shown in Raman spectra in Figure S13, both *L*/*DL*-Co-NiO samples exhibited only vibrational modes of phase-pure NiO at around 532 and 1065 cm^–1^ without any characteristic peaks of adsorbates after 1 sun irradiation, demonstrating that the photoadsorption did not critically affect PL emission peaks of Co-NiO-based catalysts.

Subsequently, to elucidate the modulation of the electronic structure in *L*-Co-NiO induced by the Co doping process, X-ray photoelectron spectroscopy (XPS) measurements were performed on *L*-Co-NiO and *L*-NiO. As shown in Figure 2e, the XPS spectra of Ni 2p_3/2_ exhibited two peaks at 853.9 and 855.5 eV, which can be attributed to Ni^2+^ in the standard Ni–O octahedral phase of the cubic rock salt structure and Ni^3+^ ions, respectively.^55,56^ The relative intensity of the Ni^3+^ peak with respect to the Ni^2+^ peak was enhanced after Co doping, indicating that *L*-Co-NiO was self-doped with Ni^3+^ ions. According to previous reports, Co atoms incorporated into the NiO lattice induce the generation of Ni^2+^ vacancy (V_Ni_) due to lattice distortions, and the high valence state of Ni^3+^ ions can be accompanied by V_Ni_.^57,58^ The NiO is a p-type semiconductor, where holes arise from the Ni^3+^ ions, which typically exist as a phase of Ni_2_O_3_.^59,60^ Furthermore, Ni^3+^ states are shallow levels near the valence band edge, resulting in a p-type self-doping.^61^ The binding energy of the Ni^2+^ 2p_3/2_ peak for *L*-Co-NiO exhibited a distinct upward shift (0.2 eV) relative to that of *L*-NiO, indicating the decrease in electron densities near the Ni^2+^ due to the increase in the Ni_2_O_3_ phase, which diminishes oxygen vacancies (i.e., electron donor).^62−64^ The O 1s XPS spectra of *L*-Co-NiO included three peaks at around 529.2, 531.0, and 532.1 eV, which correspond to the lattice oxygen involved in NiO (O_L_), oxygen in Ni_2_O_3_ (Ni_2_O_3_), and the hydroxide state of NiOOH (OH), respectively (Figure 2f).^65^ Compared with *L*-NiO, *L*-Co-NiO displayed stronger peaks representing Ni_2_O_3_ and the hydroxide state. This can be explained as follows: the Co doping produces additional Ni^3+^ states, which readily contact the water molecules at the surface, leading to the formation of the NiOOH phase. The NiOOH phase can serve as an effective active site for the OER, implying that Co doping can improve the catalytic activity of NiO. Moreover, the lattice oxygen peak of *L*-Co-NiO exhibited a positive shift of 0.16 eV with respect to that of *L*-NiO due to the decrease in oxygen vacancies; this concurs with the results in Figure 2e. In addition, the UPS analysis of *L*-NiO was also carried out to elucidate the effect of Co doping on the Fermi level shift (Figure S14). Based on the result of UPS analysis, the *E*_cuttoff_ of *L*-NiO was 16.59 eV; thus, the *E*_f_ of *L*-NiO was −4.60 eV, which was higher than that of *L*-Co-NiO (−4.79 eV). Moreover, the *E*_edge_ of *L*-NiO was 0.86 eV, indicating that the *L*-NiO exhibited a lower *E*_V_ (−5.56 eV) compared to the *L*-Co-NiO bearing the *E*_V_ of −5.46 eV. This result demonstrates that the *E*_f_ of *L*-Co-NiO was shifted near the valence band relative to that of *L*-NiO, indicative of p-type doping. In the Co 2p spectra of L-Co-NiO (Figure S15), the peaks detected at 797.4, 795.0, 782.2, and 779.6 eV correspond to the Co^3+^ 2p_1/2_, Co^2+^ 2p_1/2_, Co^3+^ 2p_3/2_, and Co^2+^ 2p_3/2_ peaks of Co-incorporated NiO, respectively^66^; this verifies that the Co atoms were successfully incorporated into the NiO lattice.

**Figure 3:**
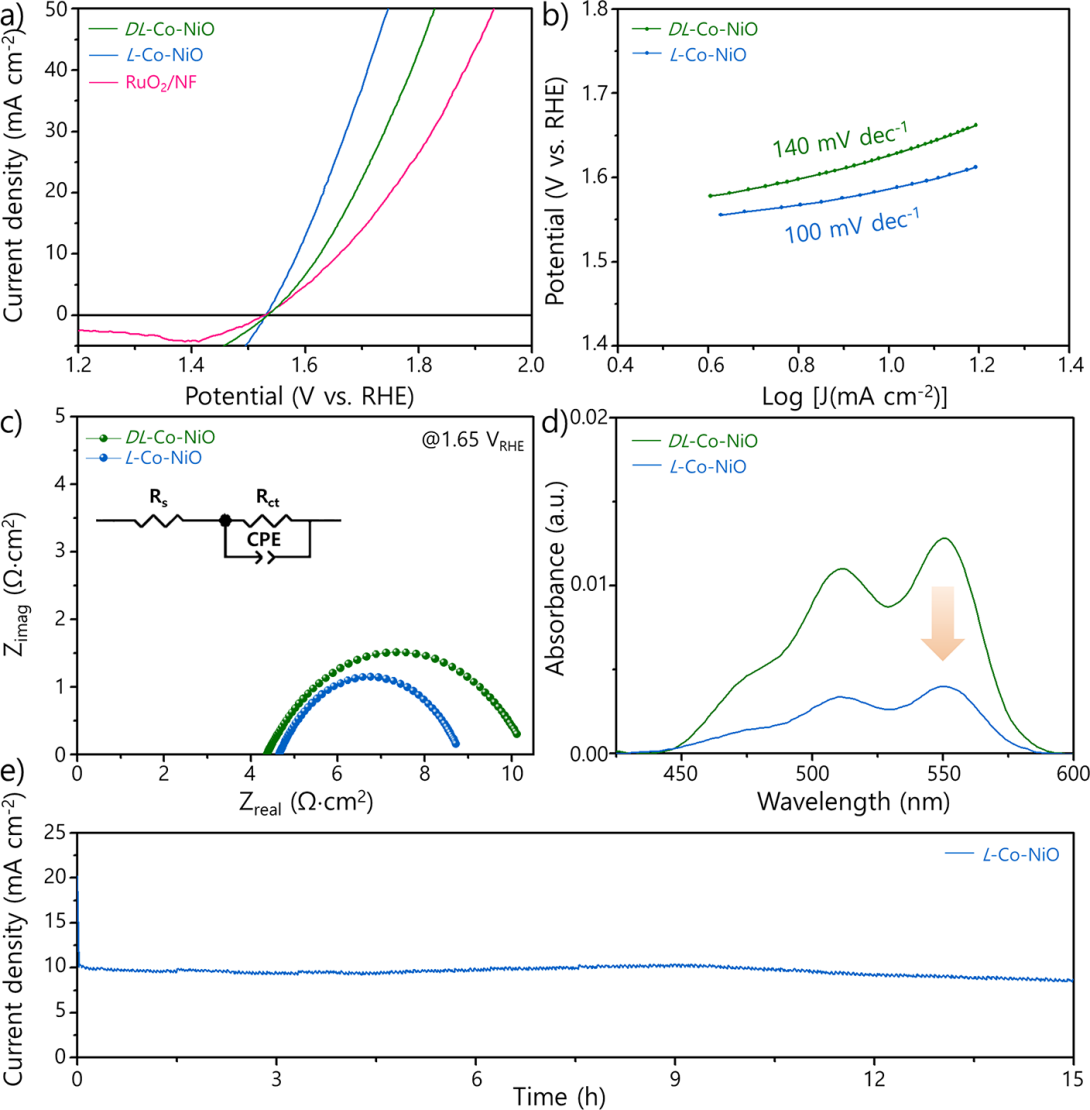
(a) LSV curves for *L*/*DL*-Co-NiO on the NF and RuO_2_/NF in a 0.2 M KOH electrolyte and (b) corresponding Tafel slopes. (c) Nyquist plots for *L*/*DL*-Co-NiO on the NF in a 0.2 M KOH electrolyte. (d) UV–vis absorption spectra of H_2_O_2_ in a 1 M KPi buffer electrolyte after OER catalysis of *L*/*DL*-Co-NiO on the NF. (e) Long-term stability of *L*-Co-NiO on the NF under the OER.

The OER electrocatalytic activities of *L*/*DL*-Co-NiO on the NF were estimated in a 0.2 M KOH electrolyte (pH ∼ 13) using a conventional three-electrode system. The forward and backward linear-sweep voltammetry (LSV) scans of *L*/*DL*-Co-NiO exhibited typical oxidation and reduction peaks, which can be ascribed to the conversion of NiO into NiOOH and the reverse reaction, respectively (Figure S16).^67^ In this regard, a backward LSV scan was conducted to clarify the OER activity of the Co-NiO-based catalysts in the absence of oxidation peaks. The LSV curves in Figure 3a indicate that the potentials required to deliver an oxygen evolution current density of 10 mA cm^–2^ were 1.58 and 1.63 V_RHE_ for *L*-Co-NiO and *DL*-Co-NiO, respectively. This result highlights a notable improvement in OER catalytic activity owing to the chirality-induced spin polarization. Moreover, the OER catalytic capability of commercial RuO_2_ particles deposited on the NF (denoted as RuO_2_/NF) was estimated, showing that *L*/*DL*-Co-NiO exhibited superior OER performance compared to RuO_2_/NF. The OER activity of *D*-Co-NiO on the NF was also investigated. Based on the findings, the potential required for achieving a current density of 10 mA cm^–2^ was 1.61 V_RHE_, which is larger than that for *L*-Co-NiO (Figure S17); this is attributed to the lower *P*_spin_ of *D*-Co-NiO compared with that of *L*-Co-NiO. The Tafel slopes of *L*-Co-NiO and *DL*-Co-NiO toward the OER were calculated to be 100 and 140 mV dec^–1^, respectively, which reflects the enhanced electrochemical reaction kinetics of *L*-Co-NiO compared with that of *DL*-Co-NiO (Figure 3b). Electrochemical impedance spectroscopy (EIS) was conducted at a potential of 1.65 V_RHE_ to scrutinize the charge transfer behavior during the OER for the *L*/*DL*-Co-NiO samples (Figure 3c). Subsequently, a Randles–Ershler circuit model consisting of series resistance (*R*_s_), charge transfer resistance (*R*_ct_), and a constant-phase element (CPE) was employed to fit the EIS analysis data (inset of Figure 3c). The Nyquist plots of the two catalysts exhibited a single semicircle at low frequencies, which corresponds to the *R*_ct_ originating from the electrocatalytic activity toward the OER. The fitted results in Table S4 verify that *L*-Co-NiO possessed a smaller *R*_ct_ than *DL*-Co-NiO, indicating the superior OER catalytic capability of the former; this is consistent with the LSV measurements.

Electrochemical active surface area (ECSA) is regarded as a critical parameter associated with the electrocatalytic activity of a material. Accordingly, to evaluate the ECSA of the *L*/*DL*-Co-NiO catalysts, their cyclic voltammetry (CV) curves were obtained at scan rates of 10–50 mV s^–1^ (Figure S18a,b). Measurements of the double-layer capacitance (*C*_dl_), which is correlated with ECSA, indicated that the *C*_dl_ of *L*-Co-NiO (27.5 mF cm^–2^) was lower than that of *DL*-Co-NiO (33.5 mF cm^–2^), as shown in Figure S18c,d. This is because the microstructure of *L*-Co-NiO comprised larger nanoplates with a lower density relative to that of *DL*-Co-NiO (Figure 1a,c). In addition, to assess the inherent OER activities of the two catalysts, their specific activity (*J*_s_)—defined as the specific current density per ECSA—was calculated using the following equations:
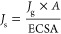
4
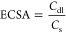
5where *J*_g_ is the current density per geometric area of the electrode, *A* represents the geometric area of *L*/*DL*-Co-NiO, and *C*_s_ is the specific capacitance, which has the same value for both samples because the catalysts were used in identical electrolytes.^16^ Interestingly, the *J*_s_ of *L*-Co-NiO was higher than that of *DL*-Co-NiO, as seen in Figure S19. These observations demonstrate that the spin polarization originating from the CISS effect can significantly elevate the intrinsic catalytic activity for the OER.

To verify that the spin alignment suppresses the generation of singlet byproducts, we investigated the formation of H_2_O_2_, a representative singlet byproduct of the OER, during the electrocatalysis of *L*/*DL*-Co-NiO on the NF. Notably, alkaline electrolytes with high pH values, such as KOH-based electrolytes, are inappropriate for the stabilization and quantification of H_2_O_2_.^23,27^ Thus, the oxygen evolution electrocatalysis was performed in a 1 M KPi buffer electrolyte (pH ∼ 6.5). As in the 0.2 M KOH electrolyte, an evident improvement in the OER catalytic ability of *L*-Co-NiO over that of *DL*-Co-NiO was observed even in the aforementioned 1 M KPi buffer electrolyte, as illustrated in Figure S20a. Subsequently, bulk electrolysis was conducted in coulometry mode with a charge of 15 C at a fixed potential of 2.0 V_RHE_ since the potential required for electrochemical H_2_O_2_ generation is 1.76 V_RHE_ (Figure S20b).^27^ More charge was consumed in *L*-Co-NiO than in *DL*-Co-NiO over the same period, owing to the superior OER activity of the former. The H_2_O_2_ produced over the two catalysts was estimated through ultraviolet–visible (UV–vis) spectrum-based colorimetric measurement after electrolysis with an equivalent charge consumed (see the Experimental Section for details). Specifically, the concentration of the electrochemically generated H_2_O_2_ was detected based on a calibration curve for the absorption peak at around 551 nm.^68^ As seen in Figure 3d, the absorption peak observed at 551 nm for *L*-Co-NiO was noticeably weaker than that for *DL*-Co-NiO, which indicates that the formation of H_2_O_2_ was suppressed owing to the chirality of *L*-Co-NiO.

In addition to the CISS effect, the Co-doping process also enhanced the OER activity of *L*-Co-NiO. For comparison, the oxygen evolution catalytic ability of *L*-NiO decorated on the NF was also analyzed in the 0.2 M KOH electrolyte (Figure S21). *L*-NiO achieved a current density of 10 mA cm^–2^ at a potential of 1.62 V_RHE_, which is 41 mV larger than that of *L*-Co-NiO. The EIS measurement was also conducted for *L*-NiO at the potential of 1.65 V_RHE_, as shown in Figure S22 and Table S5; based on the results, the *R*_ct_ of *L*-Co-NiO was less than that of *L*-NiO. This finding suggests that Co doping can improve the electrochemical OER kinetics of NiO. Furthermore, the ECSA of *L*-NiO was determined by measuring the *C*_dl_ value from the CV curve (Figure S23a,b); based on the results, the *C*_dl_ of *L*-NiO (22.5 mF cm^–2^) was smaller than that of *L*-Co-NiO (27.5 mF cm^–2^). This is because the Co doping increased the amounts of the NiOOH phase, which serves as active sites for the OER. Subsequently, a comparison of *J*_s_ between *L*-NiO and *L*-Co-NiO indicated that the intrinsic OER efficiency was improved after Co incorporation (Figure S23c). This is attributed to the enhanced hole concentration, as evidenced by the XPS results (Figure 2e,f). An improved hole concentration was also confirmed by the Mott–Schottky plot, which is generally employed to determine the acceptor concentration (*N*_A_) (Figure S24).^39^ The *L*-Co-NiO and *L*-NiO electrodes exhibited a negative slope, which reflects the p-type semiconductor characteristics of NiO. The incorporation of Co raised the *N*_A_ from 2.39 × 10^19^ to 4.29 × 10^19^ cm^–3^, thereby increasing the electrical conductivity. These results demonstrate that Co doping improves not only the density of active sites but also the inherent OER catalytic activity of NiO by increasing the hole concentration. Moreover, the long-term durability of the *L*-Co-NiO catalyst under prolonged OER was estimated in the 0.2 M KOH electrolyte (Figure 3e). The catalyst maintained nearly 90% of its initial current density after 15 h of the chronoamperometric test, which verifies its superior durability under extended OER in alkaline electrolytes.

**Figure 4:**
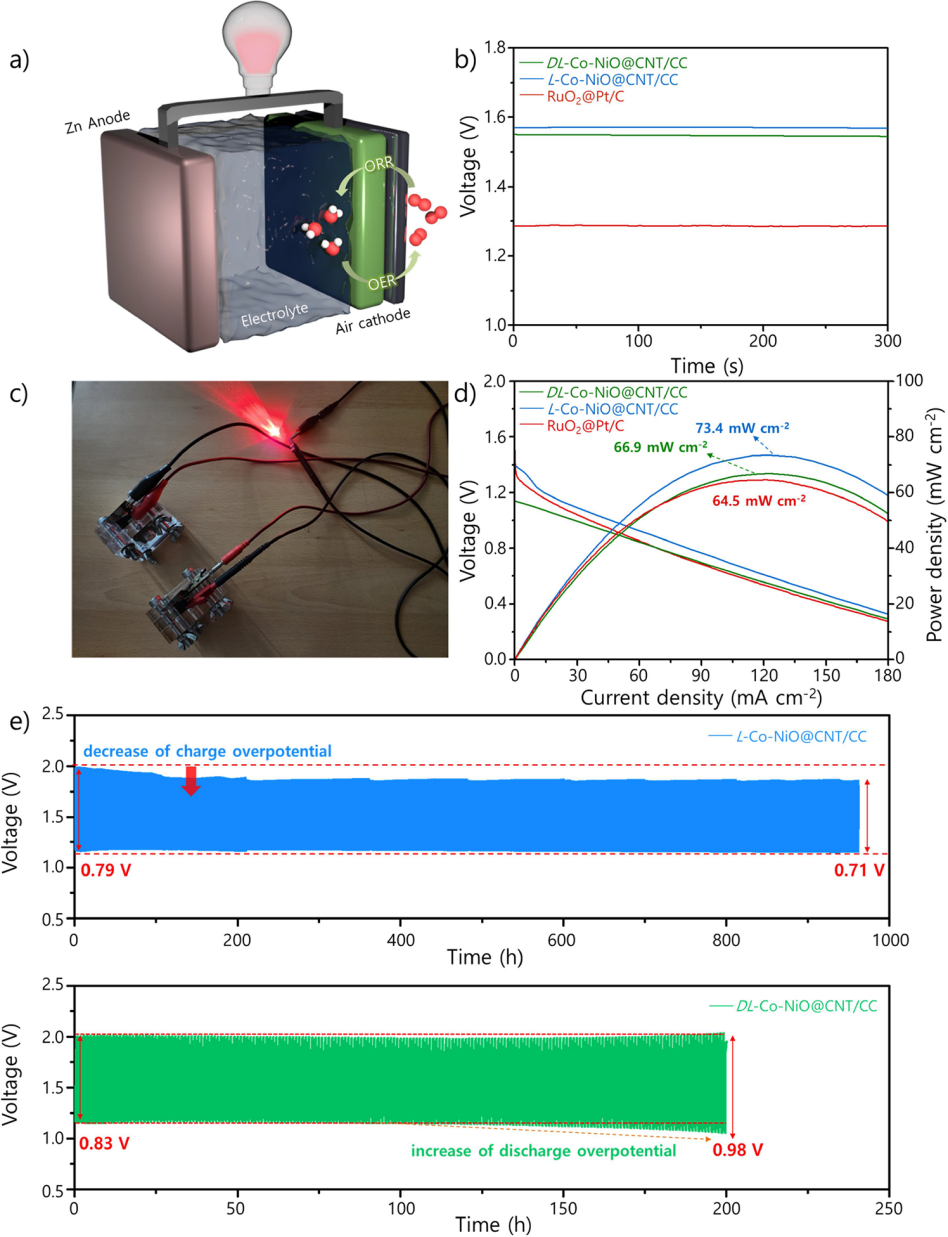
(a) Schematic of ZAB operation. (b) Open-circuit potential of *L*/*DL*-Co-NiO@CNTCC-based and RuO_2_@Pt/C-based ZABs. (c) Photograph of red LEDs powered by two *L*-Co-NiO@CNT/CC-based ZABs. (d) Power density of *L*/*DL*-Co-NiO@CNTCC-based and RuO_2_@Pt/C-based ZABs. (e) Long-term cycling curve for *L*/*DL*-Co-NiO@CNTCC-based ZABs.

Next, the efficiency of the OER performance of *L*-Co-NiO in a practical rechargeable ZAB was investigated. As illustrated in Figure 4a, the ZAB was composed of an air cathode containing *L*-Co-NiO, a highly pure Zn sheet as the anode, and a 6.0 M KOH solution with 0.2 M zinc acetate as the electrolyte. To enable both effective OER and oxygen reduction reaction (ORR) at the air electrode, the *L*-Co-NiO directly grown on the porous NF was assembled with an acid-treated carbon nanotube (CNT)-coated carbon cloth (CNT/CC) (Figure S25). As seen in Figure S26a, *L*-Co-NiO exhibited a higher current density than *DL*-Co-NiO during an ORR measurement in an O_2_-saturated 0.1 M KOH solution. This is because the ^3^O_2_ (i.e., reactant of ORR) has two unpaired electrons, which enable it to selectively accept spin-aligned electrons during the ORR, according to the Pauli exclusion principle.^69,70^ However, the overall ORR performance of *L*-Co-NiO was insufficient to implement a practical ZAB. In sharp contrast, the CNT/CC achieved a higher limiting current density of 7.9 mA cm^–2^ at a reasonable onset potential of 0.80 V, which can be attributed to the high electrical conductivity of CNT and facile adsorption of O_2_ on the CNT surface (Figure S26b).^71^ Furthermore, Figure S26c shows that the CNT/CC stably maintained a high current density of 6.6 mA cm^–2^ for over 15 h under the OER, which highlights its long-term durability. Figure S27a presents the LSV curve for CNT/CC, which was obtained after purging the electrolyte with Ar in the absence of O_2_. In this electrolyte, the CNT/CC afforded a negligible current density, highlighting that the current density observed in Figure S26b,c originates mainly from the reduction of O_2_ on the CNT/CC. Additionally, as observed in Figure S27b, the CNT/CC exhibited inferior OER performance relative to *L*-Co-NiO, indicating the necessity of simultaneously utilizing *L*-Co-NiO and CNT/CC as the OER and ORR catalysts, respectively. Therefore, based on these comprehensive results, we fabricated the air cathode by combining *L*-Co-NiO with the CNT/CC (denoted as *L*-Co-NiO@CNT/CC) to enhance both OER and ORR performance simultaneously (Figure S25).

As shown in Figure 4b, the open-circuit voltage of the *L*-Co-NiO@CNT/CC-based ZAB reached 1.57 V, which exceeds that of the achiral counterpart (1.55 V) owing to the superior electrocatalytic ability of the chiral-mesostructured Co-NiO. Notably, the ZAB with the *L*-Co-NiO@CNT/CC also exhibited a much higher open-circuit voltage than the ZAB with commercial RuO_2_@Pt/C catalysts (1.28 V); this highlights the synergistic effects of CISS and doping on enhancing ZAB performance. Moreover, two *L*-Co-NiO@CNT/CC-based ZABs connected in series could power a red light-emitting diode (LED), as displayed in Figure 4c, which underscores the potential of the proposed configuration for practical applications. Subsequently, we measured the power density of the as-prepared ZABs, observing a clear difference resulting from the chirality of the catalyst. As depicted in Figure 4d, the *L*-Co-NiO@CNT/CC-based ZAB achieved a higher power density (73.4 mW cm^–2^) than the *DL*-Co-NiO@CNT/CC-based ZAB (66.9 mW cm^–2^). The improvement can be explained by the CISS-induced spin polarization of *L*-Co-NiO, which significantly enhances the electrocatalytic activity. Meanwhile, the ZAB with the RuO_2_@Pt/C catalysts exhibited a much lower power density of 64.5 mW cm^–2^, which emphasizes the importance of the CISS effect for achieving an advanced ZAB with the highly active Co-NiO. Moreover, the *L*-Co-NiO@CNT/CC-based ZAB also delivered a higher specific capacity of 756 mA h g_Zn_^–1^, which is close to the theoretical specific capacity of ZAB (≈820 mA h g_Zn_^–1^), compared to that of the *DL*-Co-NiO@CNT/CC-based ZAB (616 mA h g_Zn_^–1^) (Figure S28). The outstanding electrochemical properties of the *L*-Co-NiO@CNT/CC-based ZAB were also confirmed through long-term cycling tests conducted at a current density of 5 mA cm^–2^. The voltage gap between the charge and discharge profiles of ZAB was calculated to be 0.79 V at the initial cycle (Figure 4e). Remarkably, the charge overpotential, which is closely related to the OER activity of air electrodes, started gradually decreasing after a few cycles, while the discharge voltage, which is associated with the ORR activity of air electrodes, remained constant; this resulted in a reduced voltage gap of 0.71 V after 960 h. In contrast, the change in the charge overpotential for the *DL*-Co-NiO@CNT/CC-based ZAB was negligible, whereas its discharge overpotential increased after 200 h; this significantly increased the voltage gap from 0.83 to 0.98 V. Moreover, the ZAB with the RuO_2_@Pt/C catalysts suffered severe degradation and could function for only 18 h (Figure S29).

**Figure 5:**
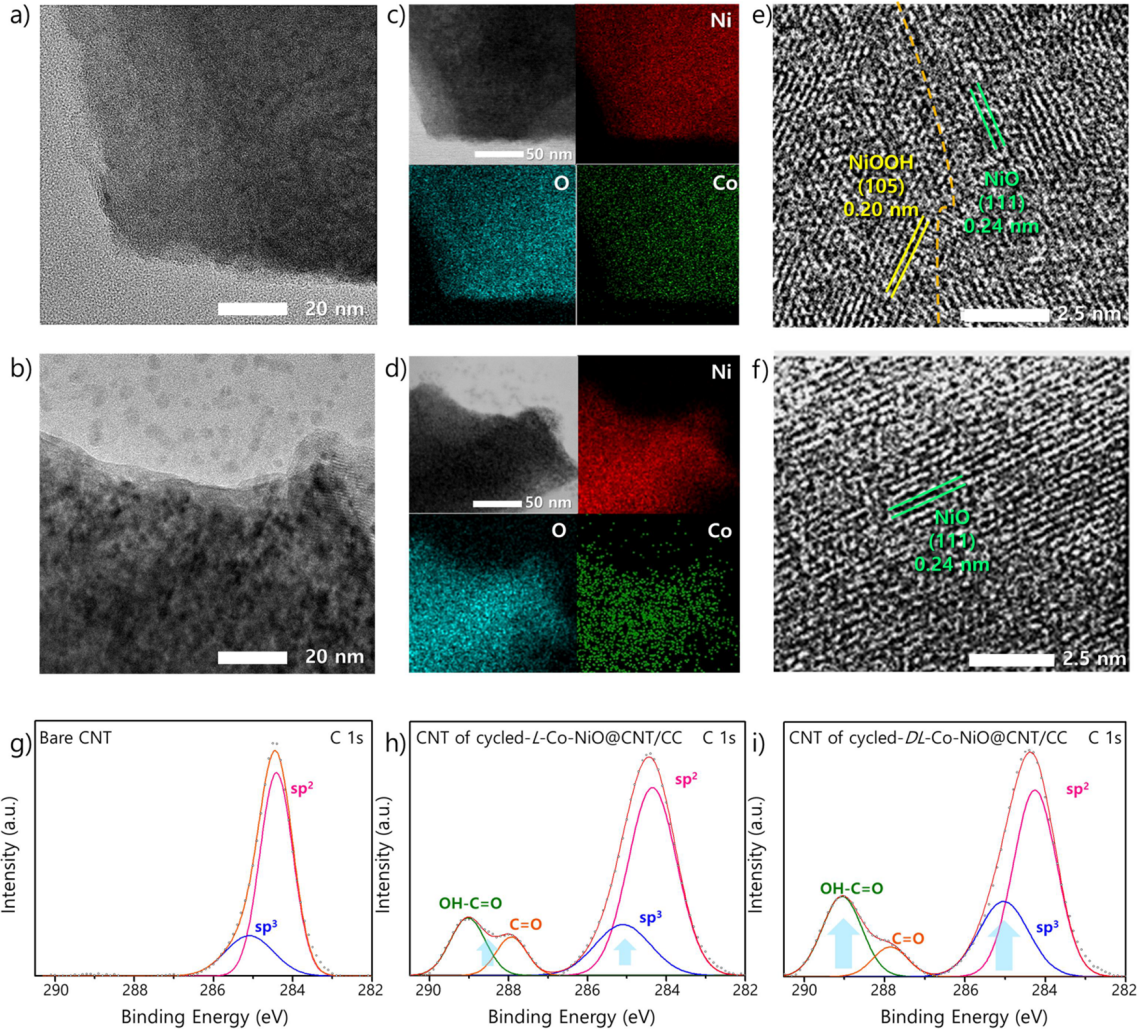
TEM images of (a) *L*-Co-NiO and (b) *DL*-Co-NiO nanoplates after cycling. STEM images and EDX mapping of (c) *L*-Co-NiO and (d) *DL*-Co-NiO nanoplates after cycling. HR-TEM images of (e) *L*-Co-NiO and (f) *DL*-Co-NiO nanoplates after cycling. High-resolution XPS spectra of (g) bare CNT, (h) CNT from cycled *L*-Co-NiO@CNT/CC, and (i) CNT from cycled *DL*-Co-NiO@CNT/CC: deconvolution of the C 1s region.

To understand the reduced overpotential of *L*-Co-NiO@CNT/CC for the charging process, we conducted additional TEM analysis on both the *L*-Co-NiO nanoplate in *L*-Co-NiO@CNT/CC and the *DL*-Co-NiO nanoplate in *DL*-Co-NiO@CNT/CC after cycling. The obtained TEM images indicate that the initial nanoplate morphologies of *L*-Co-NiO and *DL*-Co-NiO were retained even after long-term cycling (Figure 5a,b). Furthermore, the STEM images and corresponding EDX mapping data (Figure 5c,d) clarify that Ni, O, and Co atoms were still homogeneously distributed in both the *L*-Co-NiO and *DL*-Co-NiO nanoplates without any significant deterioration. We also carried out a SEM analysis for *L*/*DL*-Co-NiO on the NF after the cycling test (Figure S30). The initial nanoplate arrays of *L*/*DL*-Co-NiO on the NF were retained after the cycling test, which is consistent with TEM analysis in Figure 5a,b. Interestingly, the surfaces of nanoplate-based structures of *L*/*DL*-Co-NiO on the NF were coated with wrinkled amorphous films. This is because the crystal structure in the outer surfaces of nanoplates could be disrupted by the local stress of the significant amount of O_2_ bubbles generated by OER during the cycling test,^72^ which induced an amorphous phase on the surfaces of *L*/*DL*-Co-NiO on the NF. However, the overall nanoplate morphologies of two different catalysts were maintained, resulting in the suppression of an increase in OER overpotential during the long-term cycling tests. For a more detailed elucidation of the bulk crystal structure within the nanoplate of *L*/*DL*-Co-NiO after the cycling test, the HR-TEM analysis was performed. The HR-TEM images in Figure 5e,f show that a new NiOOH phase partially emerged on the *L*-Co-NiO nanoplate, whereas the *DL*-Co-NiO nanoplate exhibited only a NiO phase. Further, a lattice fringe with a distance of 0.20 nm was observed after cycling; this corresponds to the (105) plane of NiOOH, which can serve as favorable active sites for the OER.^73^ This result indicates that the NiO in *L*-Co-NiO was partially oxidized into NiOOH during the consecutive charging processes, which is consistent with the LSV data in Figure S16; this explains the significant reduction in the overpotential for the charging process. Conversely, the NiOOH phase on the *DL*-Co-NiO nanoplate was reduced into NiO under discharging due to the gradually increasing discharge overpotential during the cycling test; therefore, the charge overpotential of the *DL*-Co-NiO@CNT/CC-based ZAB was maintained. This increase in the overpotential for the discharge process in the achiral counterpart can be attributed to the oxidation of CNT owing to the considerable production of highly reactive ^1^O_2_ (i.e., a strong oxidizing agent for CNT) induced by the uncontrolled spin states of the electrons in *DL*-Co-NiO during the charging process (OER process).^26^ To confirm this assumption, we examined the Raman spectra of the CNT samples obtained from the cycled ZABs and determined their *I*_D_/*I*_G_ ratios. As observed in Figure S31, the CNT from the cycled *L*-Co-NiO@CNT/CC exhibited an *I*_D_/*I*_G_ ratio of 1.00, which is comparable to that of the bare CNT. By comparison, a higher ratio (1.03) was observed for the CNT from the cycled *DL*-Co-NiO@CNT/CC, implying that many defects were generated in the CNT of the achiral counterpart through parasitic reactions during cycling.^9^ XPS analysis was also performed to elucidate the chemical states of the CNT in the cycled *L*/*DL*-Co-NiO@CNT/CC. The C 1s spectra of the bare CNT exhibited two peaks at 285.0 and 284.4 eV, which are attributed to the sp^3^ carbon impurities of structural defects and the long-range ordering of the sp^2^ carbon network, respectively (Figure 5g).^74^ However, the C 1s spectra of the CNT from the cycled *L*/*DL*-Co-NiO@CNT/CC exhibited a stronger sp^3^ peak relative to that of the bare CNT, as well as additional peaks at 287.9 and 289.0 eV, corresponding to C=O and OH–C=O, respectively (Figure 5h,i).^75^ This observation suggests that after the cycling test on the ZAB, the CNT was oxidized, followed by an increase in the number of defect structures. Impressively, the intensities of the sp^3^ peak and the peak corresponding to OH–C=O, which is a more oxidative phase than C=O, were higher for the CNT from the cycled *DL*-Co-NiO@CNT/CC than for the CNT from the cycled *L*-Co-NiO@CNT/CC; this indicates that the CNT in the cycled achiral sample degraded more than that in the cycled chiral sample due to the substantial generation of ^1^O_2_ during the charging process. Such an increase in the amount of oxidative phase and the number of defect structures can reduce the electrical conductivity of CNT, resulting in reduced ORR activity and an increased discharge overpotential. Based on the aforementioned experimental results and theoretical considerations, the *L*-Co-NiO@CNT/CC-based ZAB could be stably operated over 960 h (40 days) without any significant reduction in the discharge potential, owing to the suppressed generation of ^1^O_2_ during the spin-modulated charge process induced by the CISS effect. Furthermore, considering the benefits from the accumulation of the highly active NiOOH phase, the charge overpotential was reduced during cycling, thereby reducing the voltage gap substantially. Consequently, by virtue of the spin modulation over the chiral OER catalyst, the *L*-Co-NiO@CNT/CC-based ZAB developed in this study achieved superior cycle stability compared with recently reported ZABs, without suffering any voltage gap decay (Table S6). In addition, our champion air cathode (*L*-Co-NiO@CNT/CC)-based ZAB exhibited stable operation at various current densities from 2 to 10 mA cm^–2^ (Figure S32). The *L*-Co-NiO@CNT/CC-based ZAB could achieve low overpotentials between the charge and discharge voltage of 0.30, 0.80, and 0.93 V at 2, 5, and 10 mA cm^–2^, respectively. Interestingly, the *L*-Co-NiO@CNT/CC-based ZAB showed a considerably small overpotential of 0.30 V at a current density of 2 mA cm^–2^, and this value has only been reported in a few studies.^76−79^ Moreover, when the current density returned to 2 and 5 mA cm^–2^, the values of overpotential retained 0.30 and 0.79 V, respectively, while only a slight degradation in the overpotential was observed at 10 mA cm^–2^, demonstrating the outstanding rate performance of the *L*-Co-NiO@CNT/CC-based ZAB. Based on these results, we conclude that leveraging the CISS effect by directly depositing Co-NiO on the NF represents a rational strategy for the development of next-generation ZABs.

## Conclusions

In this study, we synthesized *L*-Co-NiO as an optimized OER electrocatalyst for stable, high-performance ZABs. Owing to the chiral mesostructure of *L*-Co-NiO, the CISS effect was favorably induced in the catalyst, enabling the spin orientation of the electrons evolved during the OER to be regulated to energetically and entropically minimize the OER overpotential. The spin-modulated electrochemistry of *L*-Co-NiO was elucidated via in-depth characterizations, which demonstrated its outstanding spin selectivity and strong spin-polarization capability. In addition, the Co-doping process enhanced the electrocatalytic ability of *L*-Co-NiO further by effectively increasing both the hole conductivity and the abundance of active sites. Owing to the synergistic combination of the CISS effect and heteroatomic doping, *L*-Co-NiO exhibited superior OER performance compared with its achiral counterpart and undoped *L*-NiO. Moreover, by integrating *L*-Co-NiO with CNT/CC, which offers reasonable ORR performance, the *L*-Co-NiO@CNT/CC-based ZAB achieved a high open-circuit potential of 1.57 V, along with a low voltage gap of 0.71 V. More importantly, the considerably suppressed production of the highly corrosive ^1^O_2_ enabled the *L*-Co-NiO@CNT/CC-based ZAB to maintain excellent cycling stability for 960 h. Thus, our study provides a potentially viable route for the development of advanced ZABs by leveraging the CISS effect of Co-NiO and simultaneously improving both OER kinetics and stability of the OER from a quantum mechanical perspective.

## Experimental Section

### Preparation of Co-NiO-Based Electrodes

The nickel foam or FTO substrate (TEC-8, Pilkington, UK) was cleaned via sonification with deionized (DI) water, ethanol, and acetone for 15 min each. Then, 0.476 g of NiCl_2_·6H_2_O (Sigma-Aldrich, USA) and 0.238 g of CoCl_2_·6H_2_O (Sigma-Aldrich, 98.0%, USA) were dissolved in 40 mL of DI water to obtain a precursor solution for the hydrothermal process. Subsequently, 0.15 g of *L*-proline (Sigma-Aldrich, 99.5%, USA), *D*-proline (Sigma-Aldrich, 99.0%, USA), or racemic *DL*-proline (Sigma-Aldrich, 99.0%, USA) was added to obtain the solution for synthesizing *L*-Co-NiO, *D*-Co-NiO, or *DL*-Co-NiO, respectively. Thereafter, 2.65 mL of 25% NH_3_·H_2_O was added until the solution turned blue, following sonication for 15 min. Then, a certain amount of Co^2+^ ions could be precipitated as Co(OH)_2_. To separate the precipitate, the mixture was centrifuged at 5000 rpm for 3 min, which yielded a homogeneous supernatant solution. We used this supernatant as a precursor solution for the hydrothermal process. The hydrothermal process was performed at 140 °C for 6 h in a 100 mL Teflon-lined stainless-steel autoclave, where the cleaned nickel foam (NF) or fluorine-doped tin oxide (FTO) substrate was immersed in the precursor solution with a volume of 40 mL. The obtained electrodes were rinsed with DI water to remove the residual reactants and then dried at 60 °C for 6 h, followed by calcination at 250 °C for 3 h. The *L*-NiO was fabricated using the same method, only without the addition of CoCl_2_·6H_2_O.

### Fabrication of RuO_2_/NF

The commercial RuO_2_ powder (Sigma-Aldrich, 99.9%, USA) was decorated on a Ni foam (denoted as RuO_2_/NF) using the dispersion ink comprising 10 mg of RuO_2_ particles, 750 μL of DI water, 250 μL of isopropyl alcohol, and 20 μL of Aquivion (Sigma-Aldrich, USA). Then, the dispersion was ultrasonicated for 30 min before drop-casting onto the Ni foam with a loading of 1 mg cm^–2^ and then dried at room temperature.

### Preparation of Air Cathode for ZABs

To fabricate the air cathode for ZABs, an ink based on acid-treated CNT powders was initially prepared by adding 10 mg of CNT powder to a solution comprising 750 μL of DI water, 250 μL of isopropyl alcohol, and 150 μL of Aquivion (Sigma-Aldrich, USA). To ensure homogeneous dispersion of the carbon nanotube (CNT) powder, the ink was ultrasonicated for 30 min. The ink was then cast on carbon cloth (CC) with a loading of 1 mg cm^–2^ and dried at room temperature (denoted as CNT/CC). Subsequently, the *L*-Co-NiO@CNT/CC air electrode was fabricated by affixing *L*-Co-NiO onto the CNT/CC using Ag paste.

### Preparation of Pt/C@RuO_2_ Air Cathode

The Pt/C@RuO_2_ air cathode for the ZAB was fabricated by depositing commercial ink containing Pt/C and RuO_2_ powder on CC. This dispersion ink was prepared by adding 5 mg of platinum, nominally 20% on carbon black powder (Alfa Aesar, USA) and 5 mg of RuO_2_ particles (Sigma-Aldrich, 99.9%, USA) into a solution comprising 750 μL of DI water, 250 μL of isopropyl alcohol, and 150 μL of Aquivion (Sigma-Aldrich, USA). Then, the dispersion ink was ultrasonicated for 30 min before being cast on CC with a loading of 1 mg cm^–2^ and then dried at room temperature.

### Characterizations

The microstructures of the NiO-based electrodes were investigated through SEM (JSM-IT-500HR, JEOL, Japan). The crystallographic information on the NiO-based electrodes was obtained via XRD (Miniflex 600, Rigaku, Tokyo, Japan) with Cu K_α_ radiation (λ = 0.15406 nm). The CD and absorbance spectra of *L*/*D*/*DL*-Co-NiO on FTO were measured by using a J-815 spectrometer (JASCO Corporation, Tokyo, Japan). Raman spectroscopy of *L*/*D*/*DL*-Co-NiO on FTO and proline was performed by using a Raman microscope at an excitation wavelength of 532 nm (Alpha 300 Apyron, WITec, Germany). A commercial AM 1.5G solar simulator was employed for the Raman analysis of *L*/*D*/*DL*-Co-NiO on FTO after 1 sun irradiation for 10 min. A Si reference cell (Newport Corporation, USA) was used for 1 sun calibration. TEM (JEM-ARM200F, JEOL, Japan) analysis was performed at an acceleration voltage of 200 kV to scrutinize the morphology of the *L*/*DL*-Co-NiO nanoplate. Magnetic conductive-probe atomic force microscopy (mCP-AFM, X10, Park Systems, South Korea) was conducted using a Co–Cr-coated cantilever to evaluate the spin-polarized current of *L*/*D*/*DL*-Co-NiO on FTO. The steady-state PL analysis of *L*/*D*/*DL*-Co-NiO on FTO was conducted using a fluorescence spectrophotometer (FluoroMax-4, Horiba, Japan). The detailed band structures were elucidated by ultraviolet photoelectron spectroscopy (UPS, Nexsa G2, ThermoFisher, UK) under He I radiation (21.21 eV). The electronic structures of *L*-Co-NiO and *L*-NiO on the NF were analyzed via X-ray photoelectron spectroscopy (XPS, K-alpha, Thermo Scientific Inc., UK), and all the XPS spectra were calibrated based on the binding energy of the C 1s peak (284.4 eV).

### Electrochemical Measurements

The linear-sweep voltammetry (LSV) and chronoamperometry measurements of the NiO-based electrodes were conducted using a conventional three-electrode system comprising a Pt coil and Ag/AgCl/KCl (saturated) as the counter electrode and reference electrode, respectively. For the ORR test, the LSV and chronoamperometry measurements were performed in an O_2_-saturated electrolyte. The LSV measurement results for the NiO-based electrodes were compensated through *iR* correction. In addition, the applied potential values were converted to RHE values using the following equation:
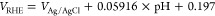
6

The EIS measurement was performed using a potentiostat with a frequency analyzer (1260, Solartron, Leicester, UK). The series resistances and polarization of the NiO-based electrodes were investigated in the frequency range of 100 kHz to 0.1 Hz with a 10 mV alternating current amplitude. The *C*_dl_ values were measured via CV to determine the ECSA. Moreover, the Mott–Schottky measurements were conducted at a frequency of 1 kHz for a range of 0–1.2 V_RHE_.

### H_2_O_2_ Detection

The *N*,*N*-diethyl-1,4-phenylene-diamine sulfate (DPD)–peroxidase (POD) method was employed to detect the electrochemically produced H_2_O_2_ in the 1 M KPi buffer electrolyte after the bulk electrolysis of *L*/*DL*-Co-NiO on the NF in coulometry mode. In brief, DPD (Sigma-Aldrich, 98.0%, USA) and POD (Sigma-Aldrich, USA) were added to 0.1 N H_2_SO_4_ (5 mL) and DI water (5 mL), respectively. Subsequently, a 0.1 M sodium phosphate buffer (2.7 mL), the DPD (0.05 mL) and POD (0.05 mL) solutions, and the 1 M KPi buffer electrolyte after the OER (0.3 mL) were mixed. The absorbance of the mixed solution at the wavelength of 551 nm was examined using a UV–vis spectrophotometer (V-670, JASCO, Easton, MD, USA).

### ZAB Assembly and Measurement

To fabricate the ZABs, the as-prepared *L*/*DL*-Co-NiO@CNT/CC or RuO_2_@Pt/C was directly used as the air electrode, a highly pure Zn sheet (0.1 mm, MTI Korea, South Korea) was used as the anode, and a 6.0 M KOH solution with 0.2 M zinc acetate was used as the electrolyte. The electrochemical performance of the ZABs was investigated by using an automatic battery cycler (WBCS 3000, WonAtech, South Korea).

## Supporting Information Available

Experimental procedure; SEM images and XRD patterns of L/D/DL-Co-NiO; TEM analysis of L-Co-NiO; Mott–Schottky (MS) plots of L-Co-NiO and L-NiO; CV curves of L/DL-Co-NiO and L-NiO; Raman spectra; XPS spectra; gas chromatography analysis; raw data of CISS analysis; LSV curves for the OER enabled by L/DL-Co-NiO in neutral electrolytes; and LSV curve for the ORR operated by CNT/CC and a comparison performance of ZABs (PDF)

## References

[ref1] Grey C. P.; Tarascon J. M. Sustainability and monitoring in battery development. Nat. Mater. 2017, 16 (1), 45–56. 10.1038/nmat4777. 27994251

[ref2] Zhang Q.; Dong S.; Shao P.; Zhu Y.; Mu Z.; Sheng D.; Zhang T.; Jiang X.; Shao R.; Ren Z.; et al. Covalent organic framework-based porous ionomers for high-performance fuel cells. Science 2022, 378 (6616), 181–186. 10.1126/science.abm6304. 36228000

[ref3] Yang X.; Su F.; Hou M.; Zhang D.; Dai Y.; Liang F. Plasma tailored reactive nitrogen species in MOF derived carbon materials for hybrid sodium–air batteries. Dalton Trans. 2021, 50 (20), 7041–7047. 10.1039/D1DT00807B. 33949530

[ref4] Su F.; Qiu X.; Liang F.; Tanaka M.; Qu T.; Yao Y.; Ma W.; Yang B.; Dai Y.; Hayashi K.; Watanabe T. Preparation of Nickel Nanoparticles by Direct Current Arc Discharge Method and Their Catalytic Application in Hybrid Na-Air Battery. Nanomaterials 2018, 8 (9), 684 10.3390/nano8090684. 30200451 PMC6165376

[ref5] Yang X.; Peng C.; Hou M.; Zhang D.; Yang B.; Xue D.; Lei Y.; Liang F. Rational Design of Electrolyte Solvation Structures for Modulating 2e^–^/4e^–^ Transfer in Sodium–Air Batteries. Adv. Funct. Mater. 2022, 32 (23), 2201258 10.1002/adfm.202201258.

[ref6] Lee D. U.; Xu P.; Cano Z. P.; Kashkooli A. G.; Park M. G.; Chen Z. Recent progress and perspectives on bi-functional oxygen electrocatalysts for advanced rechargeable metal–air batteries. J. Mater. Chem. A 2016, 4 (19), 7107–7134. 10.1039/C6TA00173D.

[ref7] Khodayar N.; Noori A.; Rahmanifar M. S.; Moloudi M.; Hassani N.; Neek-Amal M.; El-Kady M. F.; Mohamed N. B.; Xia X. H.; Zhang Y. Q.; et al. An ultra-high mass-loading transition metal phosphide electrocatalyst for efficient water splitting and ultra-durable zinc-air batteries. Energy Environ. Sci. 2024, 17 (14), 5200–5215. 10.1039/D4EE00042K.

[ref8] Liu Y. Y.; Liu S. L.; Zhang P. X.; Zhou J. J.; Liu H.; Li S. Q.; Li X.; Wang X. P.; Han D. D.; Chen Y.; et al. Electronic Structure Regulation of MnCo_2_O_4_ via Surface-Phosphorization Coupling to Monolithic Carbon for Oxygen Electrocatalysis in Zn-Air Batteries. Adv. Funct. Mater. 2024, 34 (1), 2400522 10.1002/adfm.202400522.

[ref9] Wang H. N.; Niu X. X.; Liu W. X.; Yin R. L.; Dai J. L.; Guo W.; Kong C.; Ma L.; Ding X.; Wu F. F.; et al. S-Block Metal Mg-Mediated Co—N—C as Efficient Oxygen Electrocatalyst for Durable and Temperature-Adapted Zn-Air Batteries. Adv. Sci. 2024, 34 (5), 2403865 10.1002/advs.202403865. PMC11425636 38965796

[ref10] Yang L. L.; He R.; Botifoll M.; Zhang Y. C.; Ding Y.; Di C.; He C. S.; Xu Y.; Balcells L.; Arbiol J.; et al. Enhanced Oxygen Evolution and Zinc-Air Battery Performance via Electronic Spin Modulation in Heterostructured Catalysts. Adv. Mater. 2024, 36 (31), 2400572 10.1002/adma.202400572. 38794833

[ref11] Qian J. M.; Zhang H.; Li G. Y.; Jia L.; Peng X. B.; Zhong C. L.; Li F.; Chao D. L.; Gao D. Q. Magnetic Field Modulated Intrinsic Charge and Spin Ordering in Ferromagnetic Electrocatalysts for Rechargeable Zn-Air Battery. Adv. Funct. Mater. 2024, 34 (5), 2305621 10.1002/adfm.202305621.

[ref12] Krishna B. N. V.; Ankinapalli O. R.; Reddy A. R.; Yu J. S. Strong Carbon Layer-Encapsulated Cobalt Tin Sulfide-Based Nanoporous Material as a Bifunctional Electrocatalyst for Zinc-Air Batteries. Small 2024, 20 (32), 2311176 10.1002/smll.202311176. 38528437

[ref13] Qiao J. Y.; Lu C. J.; Kong L. Q.; Zhang J.; Lin Q. Y.; Huang H. B.; Li C. F.; He W.; Zhou M.; Sun Z. M. Spin Engineering of Fe—N—C by Axial Ligand Modulation for Enhanced Bifunctional Oxygen Catalysis. Adv. Funct. Mater. 2024, 2409794 10.1002/adfm.202409794.

[ref14] Hu F.; Zhu S. L.; Chen S. M.; Li Y.; Ma L.; Wu T. P.; Zhang Y.; Wang C. M.; Liu C. C.; Yang X. J.; et al. Amorphous Metallic NiFeP: A Conductive Bulk Material Achieving High Activity for Oxygen Evolution Reaction in Both Alkaline and Acidic Media. Adv. Mater. 2017, 29 (32), 1606570 10.1002/adma.201606570. 28639333

[ref15] Zhu J.; Sun M.; Liu S. J.; Liu X. H.; Hu K.; Wang L. Study of active sites on Se-MnS/NiS heterojunctions as highly efficient bifunctional electrocatalysts for overall water splitting. J. Mater. Chem. A 2019, 7 (47), 26975–26983. 10.1039/C9TA10860B.

[ref16] Park Y. S.; Jang G.; Sohn I.; Lee H. Y. S.; Tan J.; Yun J.; Ma S.; Lee J.; Lee C. U.; Moon S.; et al. Efficient solar fuel production enabled by an iodide oxidation reaction on atomic layer deposited MoS_2_. Carbon Energy 2023, 5 (12), e366 10.1002/cey2.366.

[ref17] Qu J.; Wang Z. M.; Gan W. J.; Xiao R.; Yao X. C.; Khanam Z.; Ouyang L. Z.; Wang H.; Yang H.; Zhang S. G.; et al. Efficient Hydrogen Evolution on Antiperovskite CuNCo_3_ Nanowires by Mo Incorporation and its Trifunctionality for Zn Air Batteries and Overall Water Splitting. Small 2024, 20 (1), 2304541 10.1002/smll.202304541. 37661573

[ref18] Yun J.; Park Y. S.; Lee H.; Jeong W.; Jeong C. S.; Lee C. U.; Lee J.; Moon S.; Kwon E.; Lee S.; et al. Efficient and Ultrastable Iodide Oxidation Reaction Over Defect-Passivated Perovskite Photoanode for Unassisted Solar Fuel Production. Adv. Energy Mater. 2024, 14, 2401055 10.1002/aenm.202401055.

[ref19] Sanchez J. S.; Xia Z. Y.; Mirehbar K.; Sasidharan S.; Aravindh S. A.; Liscio A.; Sun J. H.; Christian M.; Palma J.; Palermo V.; et al. Versatile electrochemical manufacturing of mixed metal sulfide/N-doped rGO composites as bifunctional catalysts for high power rechargeable Zn-air batteries. J. Mater. Chem. A 2024, 12 (20), 11945–11959. 10.1039/D3TA07765A.

[ref20] Behera A.; Seth D.; Agarwal M.; Haider M. A.; Bhattacharyya A. J. Exploring Cu-Doped Co_3_O_4_ Bifunctional Oxygen Electrocatalysts for Aqueous Zn-Air Batteries. ACS Appl. Mater. Interfaces 2024, 16 (14), 17574–17586. 10.1021/acsami.4c00571. 38556732

[ref21] Chae K.; Mohamad N. A. R. C.; Kim J.; Won D. I.; Lin Z. Q.; Kim J.; Kim D. H. The promise of chiral electrocatalysis for efficient and sustainable energy conversion and storage: a comprehensive review of the CISS effect and future directions. Chem. Soc. Rev. 2024, 53, 9029–9058. 10.1039/D3CS00316G. 39158537

[ref22] Lee H.; Lee C. U.; Yun J.; Jeong C. S.; Jeong W.; Son J.; Park Y. S.; Moon S.; Lee S.; Kim J. H.; et al. A dual spin-controlled chiral two-/three-dimensional perovskite artificial leaf for efficient overall photoelectrochemical water splitting. Nat. Commun. 2024, 15 (1), 4672 10.1038/s41467-024-49216-x. 38824151 PMC11144254

[ref23] Bian Z. Y.; Kato K.; Ogoshi T.; Cui Z.; Sa B. S.; Tsutsui Y.; Seki S.; Suda M. Hybrid Chiral MoS_2_ Layers for Spin-Polarized Charge Transport and Spin-Dependent Electrocatalytic Applications. Adv. Sci. 2022, 9 (17), 2201063 10.1002/advs.202201063. PMC9189682 35481673

[ref24] Lee H.; Ma S.; Oh S.; Tan J. W.; Lee C. U.; Son J.; Park Y. S.; Yun J.; Jang G.; Moon J. Chirality-Induced Spin Selectivity of Chiral 2D Perovskite Enabling Efficient Spin-Dependent Oxygen Evolution Reaction. Small 2023, 19 (40), 2304166 10.1002/smll.202304166. 37282813

[ref25] Im H.; Ma S.; Lee H.; Park J.; Park Y. S.; Yun J.; Lee J.; Moon S.; Moon J. Elucidating the chirality transfer mechanisms during enantioselective synthesis for the spin-controlled oxygen evolution reaction. Energy Environ. Sci. 2023, 16 (4), 1797–1797. 10.1039/D3EE90020G.

[ref26] Chan S. P.; Chen G.; Gong X. G.; Liu Z. F. Oxidation of carbon nanotubes by singlet O_2_*Phys*. Rev. Lett. 2003, 90 (8), 086403 10.1103/PhysRevLett.90.086403. 12633446

[ref27] Feng T. L.; Chen W. H.; Xue J.; Cao F. F.; Chen Z. W.; Ye J. C.; Xiao C. X.; Lu H. P. Spin Polarization of Chiral Amorphous Fe-Ni Electrocatalysts Enabling Efficient Electrochemical Oxygen Evolution. Adv. Funct. Mater. 2023, 33 (27), 2215051 10.1002/adfm.202215051.

[ref28] Lee C. U.; Lee H.; Jeong C. S.; Ma S.; Jang G.; Park Y. S.; Yun J.; Lee J.; Son J.; Jeong W.; et al. Enhanced Stability of Spin-Dependent Chiral 2D Perovskite Embedded PV-Biased Anode via Cross-Linking Strategy. ACS Energy Lett. 2024, 9 (8), 4032–4043. 10.1021/acsenergylett.4c01721.

[ref29] Son J.; Jang G.; Ma S.; Lee H.; Lee C. U.; Yang S.; Lee J.; Moon S.; Jeong W.; Park J. H.; et al. Fluorinated Organic Cations Derived Chiral 2D Perovskite Enabling Enhanced Spin-Dependent Oxygen Evolution Reaction. Adv. Sci. 2024, 11 (33), 2403326 10.1002/advs.202403326. PMC11434140 38940393

[ref30] Bai T.; Ai J.; Liao L. Y.; Luo J. W.; Song C.; Duan Y. Y.; Han L.; Che S. A. Chiral Mesostructured NiO Films with Spin Polarisation. Angew. Chem., Int. Ed. 2021, 60 (17), 9421–9426. 10.1002/anie.202101069. 33554464

[ref31] Bian Z. Y.; Nakano Y.; Miyata K.; Oya I.; Nobuoka M.; Tsutsui Y.; Seki S.; Suda M. Chiral Van Der Waals Superlattices for Enhanced Spin-Selective Transport and Spin-Dependent Electrocatalytic Performance. Adv. Mater. 2023, 35 (48), 2306061 10.1002/adma.202306061. 37695880

[ref32] Liang Y. C.; Banjac K.; Martin K.; Zigon N.; Lee S.; Vanthuyne N.; Garcés-Pineda F. A.; Galán-Mascarós J. R.; Hu X. L.; Avarvari N.; et al. Enhancement of electrocatalytic oxygen evolution by chiral molecular functionalization of hybrid 2D electrodes. Nat. Commun. 2022, 13 (1), 3356 10.1038/s41467-022-31096-8. 35688831 PMC9187664

[ref33] Jin Y. R.; Fu W. L.; Wen Z. H.; Tan L. L.; Chen Z.; Wu H.; Wang P. P. Chirality Engineering of Colloidal Copper Oxide Nanostructures for Tailored Spin-Polarized Catalysis. J. Am. Chem. Soc. 2024, 146 (4), 2798–2804. 10.1021/jacs.3c12965. 38145451

[ref34] Zhang W. Y.; Banerjee-Ghosh K.; Tassinari F.; Naaman R. Enhanced Electrochemical Water Splitting with Chiral Molecule-Coated FeO Nanoparticles. ACS Energy Lett. 2018, 3 (10), 2308–2313. 10.1021/acsenergylett.8b01454.

[ref35] Creamer T. P. Left-handed polyproline II helix formation is (very) locally driven. Proteins 1998, 33 (2), 218–226. 10.1002/(SICI)1097-0134(19981101)33:2<218::AID-PROT6>3.0.CO;2-E. 9779789

[ref36] Ai M. H.; Pan L.; Shi C. X.; Huang Z. F.; Zhang X. W.; Mi W. B.; Zou J. J. Spin selection in atomic-level chiral metal oxide for photocatalysis. Nat. Commun. 2023, 14 (1), 4562 10.1038/s41467-023-40367-x. 37507418 PMC10382512

[ref37] Park Y. S.; Jin X. Y.; Tan J. W.; Lee H.; Yun J.; Ma S.; Jang G. M.; Kim T.; Shim S. G.; Kim K.; et al. High-performance Sb_2_S_3_ photoanode enabling iodide oxidation reaction for unbiased photoelectrochemical solar fuel production. Energy Environ. Sci. 2022, 15 (11), 4725–4737. 10.1039/D1EE02940A.

[ref38] Sorenson B. A.; Yoon L. U.; Holmgren E.; Choi J. J.; Clancy P. A new metric to control nucleation and grain size distribution in hybrid organic-inorganic perovskites by tuning the dielectric constant of the antisolvent. J. Mater. Chem. A 2021, 9 (6), 3668–3676. 10.1039/D0TA12364A.

[ref39] Lee H.; Yang W.; Tan J.; Oh Y.; Park J.; Moon J. Cu-Doped NiO_x_ as an Effective Hole-Selective Layer for a High-Performance Sb_2_Se_3_ Photocathode for Photoelectrochemical Water Splitting. ACS Energy Lett. 2019, 4 (5), 995–1003. 10.1021/acsenergylett.9b00414.

[ref40] Kabbur M.; Waghmare S. D.; Ghodake U. R.; Suryavanshi S. S. Synthesis, Morphology and Electrical Properties of Co^2+^ Substituted NiCuZn Ferrites for MLCI Applications. AIP Conf. Proc. 2018, 1942, 130002 10.1063/1.5029072.

[ref41] Sun Y.; Wang J.; Qi Y. F.; Li W. J.; Wang C. Efficient Electrooxidation of 5-Hydroxymethylfurfural Using Co-Doped Ni_3_S_2_ Catalyst: Promising for H_2_ Production under Industrial-Level Current Density. Adv. Sci. 2022, 9 (17), 2200957 10.1002/advs.202200957. PMC9189636 35426484

[ref42] Liu S. P.; Kepenekian M.; Bodnar S.; Feldmann S.; Heindl M. W.; Fehn N.; Zerhoch J.; Shcherbakov A.; Pöthig A.; Li Y.; et al. Bright circularly polarized photoluminescence in chiral layered hybrid lead-halide perovskites. Sci. Adv. 2023, 9 (35), eadh5083 10.1126/sciadv.adh5083. 37656792 PMC10854422

[ref43] Huang W.; Ding S. J.; Chen Y.; Hao W. J.; Lai X. Y.; Peng J.; Tu J. C.; Cao Y.; Li X. T. 3D NiO hollow sphere/reduced graphene oxide composite for high-performance glucose biosensor. Sci. Rep. 2017, 7, 5220 10.1038/s41598-017-05528-1. 28701794 PMC5507916

[ref44] Liu M.; Ji Y. J.; Li Y. Y.; An P. F.; Zhang J.; Yan J. Q.; Liu S. Z. Single-Atom Doping and High-Valence State for Synergistic Enhancement of NiO Electrocatalytic Water Oxidation. Small 2021, 17 (36), 2102448 10.1002/smll.202102448. 34323372

[ref45] Göhler B.; Hamelbeck V.; Markus T. Z.; Kettner M.; Hanne G. F.; Vager Z.; Naaman R.; Zacharias H. Spin Selectivity in Electron Transmission Through Self-Assembled Monolayers of Double-Stranded DNA. Science 2011, 331 (6019), 894–897. 10.1126/science.1199339. 21330541

[ref46] Kiran V.; Mathew S. P.; Cohen S. R.; Delgado I. H.; Lacour J.; Naaman R. Helicenes-A New Class of Organic Spin Filter. Adv. Mater. 2016, 28 (10), 1957 10.1002/adma.201504725. 26742997

[ref47] Kiran V.; Cohen S. R.; Naaman R. Structure dependent spin selectivity in electron transport through oligopeptides. J. Chem. Phys. 2017, 146 (9), 092302 10.1063/1.4966237.

[ref48] Nachammai J.; Perumal P.; Deivamani D.; Saravanakumar S. Effect of concentrations and characterization of nickel oxide thin films prepared by SILAR method. Mater. Today Proc. 2022, 64, 1789–1792. 10.1016/j.matpr.2022.06.065.

[ref49] Chen M.; Zha R. H.; Yuan Z. Y.; Jing Q. S.; Huang Z. Y.; Yang X. K.; Yang S. M.; Zhao X. H.; Xu D. L.; Zou G. D. Boron and phosphorus co-doped carbon counter electrode for efficient hole-conductor-free perovskite solar cell. Chem. Eng. J. 2017, 313 (1), 791–800. 10.1016/j.cej.2016.12.050.

[ref50] Xia J.; Yuan C. C.; Yanagida S. Novel Counter Electrode V_2_O_5_/Al for Solid Dye-Sensitized Solar Cells. ACS Appl. Mater. Interfaces 2010, 2 (7), 2136–2139. 10.1021/am100380w.

[ref51] Teng F.; Hu K.; Ouyang W.; Fang X. S. Photoelectric Detectors Based on Inorganic p-Type Semiconductor Materials. Adv. Mater. 2018, 30 (35), 1706262 10.1002/adma.201706262. 29888448

[ref52] Saha B.; Sarkar K.; Bera A.; Deb K.; Thapa R. Schottky diode behaviour with excellent photoresponse in NiO/FTO heterostructure. Appl. Surf. Sci. 2017, 418, 328–334. 10.1016/j.apsusc.2017.01.142.

[ref53] Wei Y. J.; Chang X. X.; Wang T.; Li C. C.; Gong J. L. A Low-Cost NiO Hole Transfer Layer for Ohmic Back Contact to Cu_2_O for Photoelectrochemical Water Splitting. Small 2017, 13 (39), 1702007 10.1002/smll.201702007. 28786522

[ref54] Gao W. Q.; Peng R.; Yang Y. Y.; Zhao X. L.; Cui C.; Su X. W.; Qin W.; Dai Y.; Ma Y. D.; Liu H.; et al. Electron Spin Polarization-Enhanced Photoinduced Charge Separation in Ferromagnetic ZnFe_2_O_4_. ACS Energy Lett. 2021, 6 (6), 2129–2137. 10.1021/acsenergylett.1c00682.

[ref55] Safeer N. K. M.; Alex C.; Jana R.; Datta A.; John N. S. Remarkable CO_x_ tolerance of Ni^3+^ active species in a Ni_2_O_3_ catalyst for sustained electrochemical urea oxidation. J. Mater. Chem. A 2022, 10 (8), 4209–4221. 10.1039/D1TA05753G.

[ref56] Wang S. X.; Zhang B. J.; Feng D.; Lin Z. H.; Zhang J. C.; Hao Y.; Fan X. Y.; Chang J. J. Achieving high performance and stable inverted planar perovskite solar cells using lithium and cobalt co-doped nickel oxide as hole transport layers. J. Mater. Chem. C 2019, 7 (30), 9270–9277. 10.1039/C9TC02526J.

[ref57] Yuan X.; Li H.; Fan J.; Zhang L.; Ran F.; Feng M.; Li P.; Kong W.; Chen S.; Zang Z.; Wang S. Enhanced p-Type Conductivity of NiO_x_ Films with Divalent Cd Ion Doping for Efficient Inverted Perovskite Solar Cells. ACS Appl. Mater. Interfaces 2022, 14 (15), 17434–17443. 10.1021/acsami.2c01813. 35394734

[ref58] Lee J. H.; Noh Y. W.; Jin I. S.; Park S. H.; Jung J. W. A solution-processed cobalt-doped nickel oxide for high efficiency inverted type perovskite solar cells. J. Power Sources 2019, 412 (1), 425–432. 10.1016/j.jpowsour.2018.11.081.

[ref59] Kaneko R.; Chowdhury T. H.; Wu G.; Kayesh M. E.; Kazaoui S.; Sugawa K.; Lee J.-J.; Noda T.; Islam A.; Otsuki J. Cobalt-doped nickel oxide nanoparticles as efficient hole transport materials for low-temperature processed perovskite solar cells. Sol. Energy 2019, 181 (15), 243–250. 10.1016/j.solener.2019.01.097.

[ref60] Xu R.; Xu X.; Luo R.; Li Y.; Wang G.; Liu T.; Cai N.; Yang S. D-π-D molecular layer electronically bridges the NiO*_x_* hole transport layer and the perovskite layer towards high performance photovoltaics. J. Energy Chem. 2022, 67, 797–804. 10.1016/j.jechem.2021.11.029.

[ref61] Di Girolamo D.; Phung N.; Jošt M.; Al-Ashouri A.; Chistiakova G.; Li J.; Márquez J. A.; Unold T.; Korte L.; Albrecht S.; Di Carlo A.; Dini D.; Abate A. From Bulk to Surface: Sodium Treatment Reduces Recombination at the Nickel Oxide/Perovskite Interface. Adv. Mater. Interfaces 2019, 6 (17), 1900789 10.1002/admi.201900789.

[ref62] Shim S. G.; Tan J.; Lee H.; Park J.; Yun J.; Park Y. S.; Kim K.; Lee J. Y.; Moon J. Facile morphology control strategy to enhance charge separation efficiency of Mo:BiVO_4_ photoanodes for efficient photoelectrochemical water splitting. Chem. Eng. J. 2022, 430 (15), 133061 10.1016/j.cej.2021.133061.

[ref63] Pan J. B.; Wang B. H.; Wang J. B.; Ding H. Z.; Zhou W.; Liu X.; Zhang J. R.; Shen S.; Guo J. K.; Chen L.; Au C. T.; Jiang L. L.; Yin S. F. Activity and Stability Boosting of an Oxygen-Vacancy-Rich BiVO_4_ Photoanode by NiFe-MOFs Thin Layer for Water Oxidation. Angew. Chem., Int. Ed. 2021, 60 (3), 1433–1440. 10.1002/anie.202012550. 33006403

[ref64] Elseman A. M.; Luoa L.; Song Q. L. Self-doping synthesis of trivalent Ni_2_O_3_ as a hole transport layer for high fill factor and efficient inverted perovskite solar cells. Dalton Trans. 2020, 49, 14243–14250. 10.1039/D0DT03029E. 33025991

[ref65] Liu Z.; Chang J.; Lin Z.; Zhou L.; Yang Z.; Chen D.; Zhang C.; Liu S. F.; Hao Y. High-Performance Planar Perovskite Solar Cells Using Low Temperature, Solution–Combustion-Based Nickel Oxide Hole Transporting Layer with Efficiency Exceeding 20%. Adv. Energy Mater. 2018, 8 (19), 1703432 10.1002/aenm.201703432.

[ref66] Xie H. Y.; Wang K.; Xiang D. Z.; Li S. L.; Jin Z. L. Enwrapping graphdiyne (g-C_n_H_2n-2_) on hollow NiCo_2_O_4_ nanocages derived from a Prussian blue analogue as a p-n heterojunction for highly efficient photocatalytic hydrogen evolution. J. Mater. Chem. A 2023, 11 (27), 14971–14989. 10.1039/D3TA02598E.

[ref67] Liang K.; Tang X. Z.; Hu W. C. High-performance three-dimensional nanoporous NiO film as a supercapacitor electrode. J. Mater. Chem. 2012, 22 (22), 11062–11067. 10.1039/c2jm31526b.

[ref68] Moon S.; Park Y. S.; Lee H.; Jeong W.; Kwon E.; Lee J.; Yun J.; Lee S.; Kim J. H.; Yu S.; et al. Unassisted photoelectrochemical hydrogen peroxide production over MoO_x_-supported Mo on a Cu_3_BiS_3_ photocathode. Energy Environ. Sci. 2024, 17 (15), 5588–5600. 10.1039/D4EE00741G.

[ref69] Sang Y. T.; Tassinari F.; Santra K.; Zhang W. Y.; Fontanesi C.; Bloom B. P.; Waldeck D. H.; Fransson J.; Naaman R. Chirality enhances oxygen reduction. Proc. Natl. Acad. Sci. U. S. A. 2022, 119 (30), e2202650119 10.1073/pnas.2202650119. 35858429 PMC9335305

[ref70] Gupta A.; Sang Y. T.; Fontanesi C.; Turin L.; Naaman R. Effect of Anesthesia Gases on the Oxygen Reduction Reaction. J. Phys. Chem. Lett. 2023, 14 (7), 1756–1761. 10.1021/acs.jpclett.2c03753. 36779610 PMC9940288

[ref71] Han J. R.; Shi L.; Xie H. M.; Song R. L.; Wang D.; Liu D. Self-Powered Electrochemical CO_2_ Conversion Enabled by a Multifunctional Carbon-Based Electrocatalyst and a Rechargeable Zn-Air Battery. Small 2024, 20 (40), 2401766 10.1002/smll.202401766. 38837621

[ref72] Wang J.; Liang C.; Ma X.; Liu P.; Pan W.; Zhu H.; Guo Z.; Sui Y.; Liu H.; Liu L.; Yang C. Dynamically Adaptive Bubbling for Upgrading Oxygen Evolution Reaction Using Lamellar Fern-Like Alloy Aerogel Self-Standing Electrodes. Adv. Mater. 2024, 36 (1), 2307925 10.1002/adma.202307925. 37742133

[ref73] He Z. Y.; Zhang J.; Gong Z. H.; Lei H.; Zhou D.; Zhang N. A.; Mai W. J.; Zhao S. J.; Chen Y. Activating lattice oxygen in NiFe-based (oxy)hydroxide for water electrolysis. Nat. Commun. 2022, 13 (1), 2191 10.1038/s41467-022-29875-4. 35449165 PMC9023528

[ref74] Koizhaiganova R. B.; Hwang D. H.; Lee C. J.; Roth S.; Dettlaff-Weglikowska U. N-type doping effect of single-walled carbon nanotubes with aromatic amines. Phys. Status Solidi. B 2010, 247 (11–12), 2793–2796. 10.1002/pssb.201000165.

[ref75] Kovtyukhova N. I.; Mallouk T. E.; Pan L.; Dickey E. C. Individual single-walled nanotubes and hydrogels made by oxidative exfoliation of carbon nanotube ropes. J. Am. Chem. Soc. 2003, 125 (32), 9761–9769. 10.1021/ja0344516. 12904042

[ref76] Zhang W.; Chu J.; Li S.; Li Y.; Li L. CoN*_x_*C active sites-rich three-dimensional porous carbon nanofibers network derived from bacterial cellulose and bimetal-ZIFs as efficient multifunctional electrocatalyst for rechargeable Zn–air batteries. J. Energy Chem. 2020, 51, 323–332. 10.1016/j.jechem.2020.04.067.

[ref77] Wang L.; Wang X.-T.; Zhong J.-H.; Xiao K.; Ouyang T.; Liu Z.-Q. Filling the Charge–Discharge Voltage Gap in Flexible Hybrid Zinc-Based Batteries by Utilizing a Pseudocapacitive Material. Chem.—Eur. J. 2021, 27 (18), 5796–5802. 10.1002/chem.202100112. 33491256

[ref78] Peng H.; Duan D.; Liu S.; Liu J.; Sun L.; Huang P.; Shao C.; Zhang K.; Zhang H.; Xue X.; Xu F.; Zou Y.; Liu Y.; Tian X.; Rosei F. A graphene-like nanoribbon for efficient bifunctional electrocatalysts. J. Mater. Chem. A 2021, 9 (47), 26688–26697. 10.1039/D1TA06078C.

[ref79] Chen Y.; He T.; Liu Q.; Hu Y.; Gu H.; Deng L.; Liu H.; Liu Y.; Liu Y.-N.; Zhang Y.; Chen S.; Ouyang X. Highly durable iron single-atom catalysts for low-temperature zinc-air batteries by electronic regulation of adjacent iron nanoclusters. Appl. Catal., B 2023, 323, 122163 10.1016/j.apcatb.2022.122163.

