# Supplementary material for: Elucidating the Chirality-Induced Spin Selectivity Effect of Co-Doped NiO Deposited on Ni Foam for Highly Stable Zn–Air Batteries
Source: ACS Appl Mater Interfaces. 2025 Mar 13;17(12):18228–42. doi: 10.1021/acsami.4c20630 (PMC11955944; doi:10.1021/acsami.4c20630)
Supplement: Supplementary file 1 — am4c20630_si_001.pdf [file am4c20630_si_001.pdf]

Supporting Information

Elucidating Chirality-Induced Spin Selectivity Effect of Co-doped NiO Deposited on Ni Foam for Highly Stable Zn–Air Batteries

Young Sun Park,[1†] *Jeongyoub Lee,* [1†] *Hyungsoo Lee,* [1] *Jung Been Park,*[2] *Juwon Yun,*[1]*Chan Uk Lee,*[1] *Subin Moon,*[1] *Soobin Lee*,[1] *Sumin Kim,*[1] *Junhwan Kim*,[1] *Donghyun Kim,*[1] *Jimin Han,*[1] *Dong-Wan Kim,*[2] and *Jooho Moon*[1]*

[1]Department of Materials Science and Engineering Yonsei University Seoul, 03722, Republic of Korea.

[2]School of Civil, Environmental, and Architectural Engineering, Korea University, Seoul 02841, Republic of Korea

†These authors contributed equally.

*E-mail: jmoon@yonsei.ac.kr

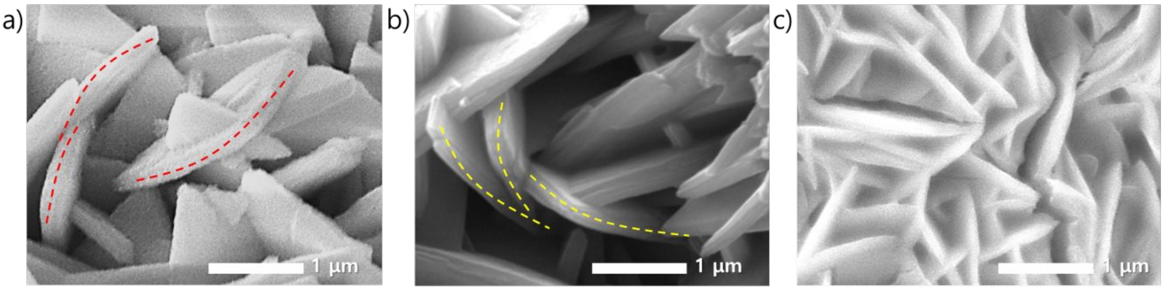

Figure S1. SEM images of a) *L*-Co-NiO, b) *D*-Co-NiO, and c) *DL*-Co-NiO on NF.

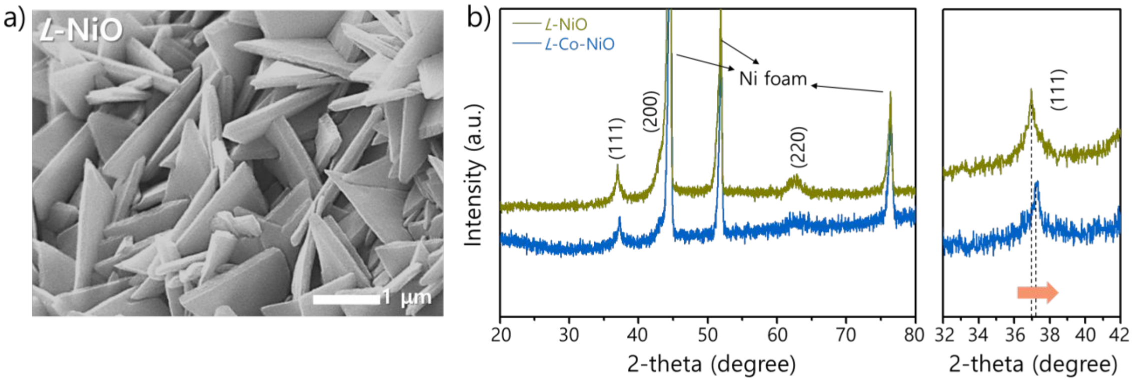

Figure S2. a) SEM image of *L*-NiO on NF. b) XRD data for *L*-Co-NiO and *L*-NiO on NF.

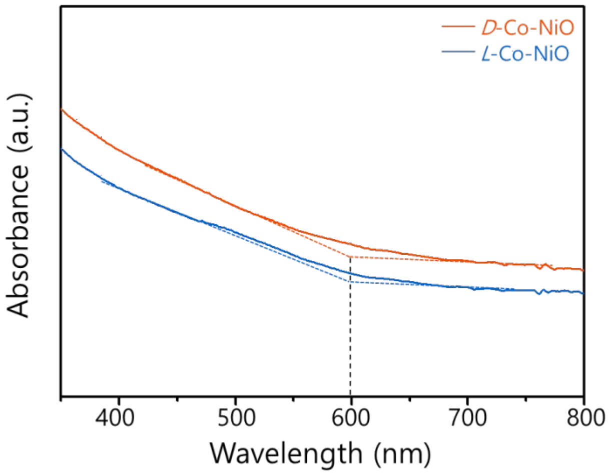

Figure S3. Absorbance of *L*/*D*-Co-NiO on FTO.

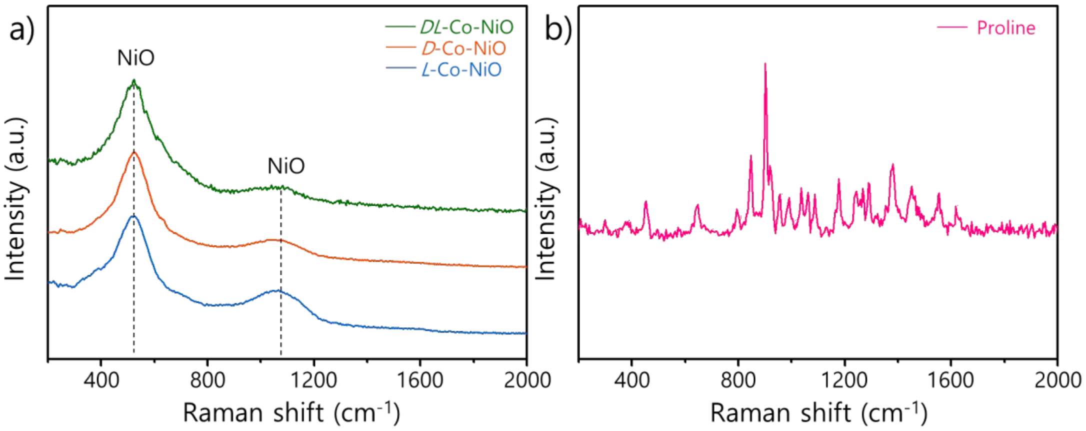

Figure S4. Raman spectra of a) *L*/*D*/*DL*-Co-NiO on FTO and b) proline.

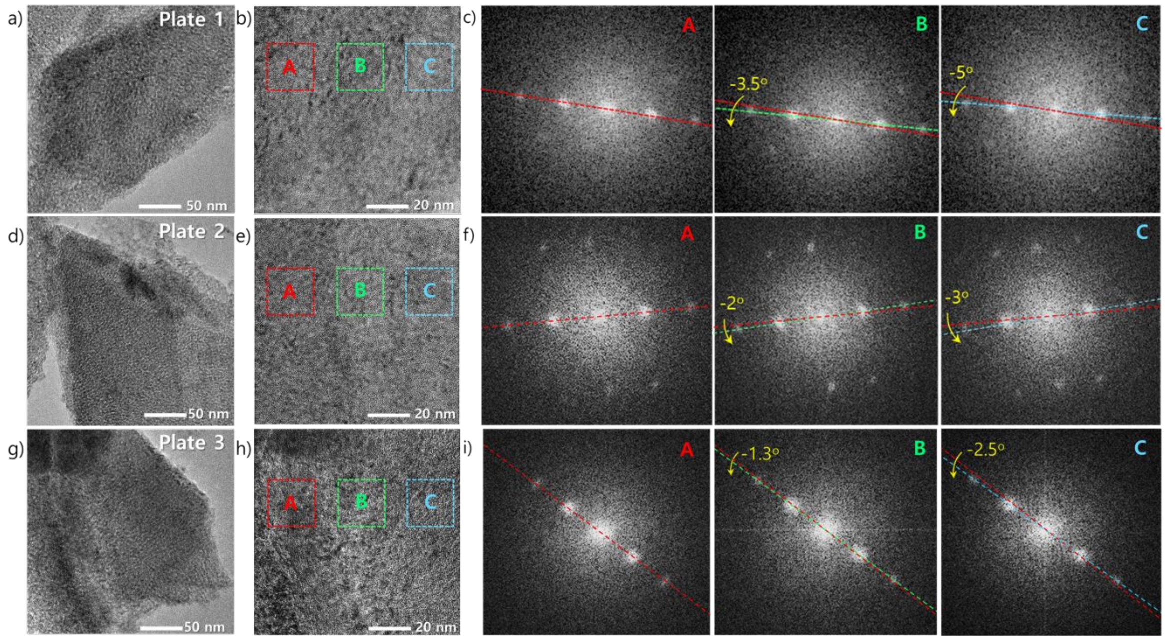

Figure S5. a) TEM image of *L*-Co-NiO nanoplate 1, b) HR-TEM image of nanoplate 1 and c) FFT images of three different positions (A, B, and C) in HR-TEM image of nanoplate 1. d) TEM image of *L*-Co-NiO nanoplate 2, e) HR-TEM image of nanoplate 2 and f) FFT images of three different positions (A, B, and C) in HR-TEM image of nanoplate 2. g) TEM image of *L*-Co-NiO nanoplate 3, h) HR-TEM image of nanoplate 3 and i) FFT images of three different positions (A, B, and C) in nanoplate 3.

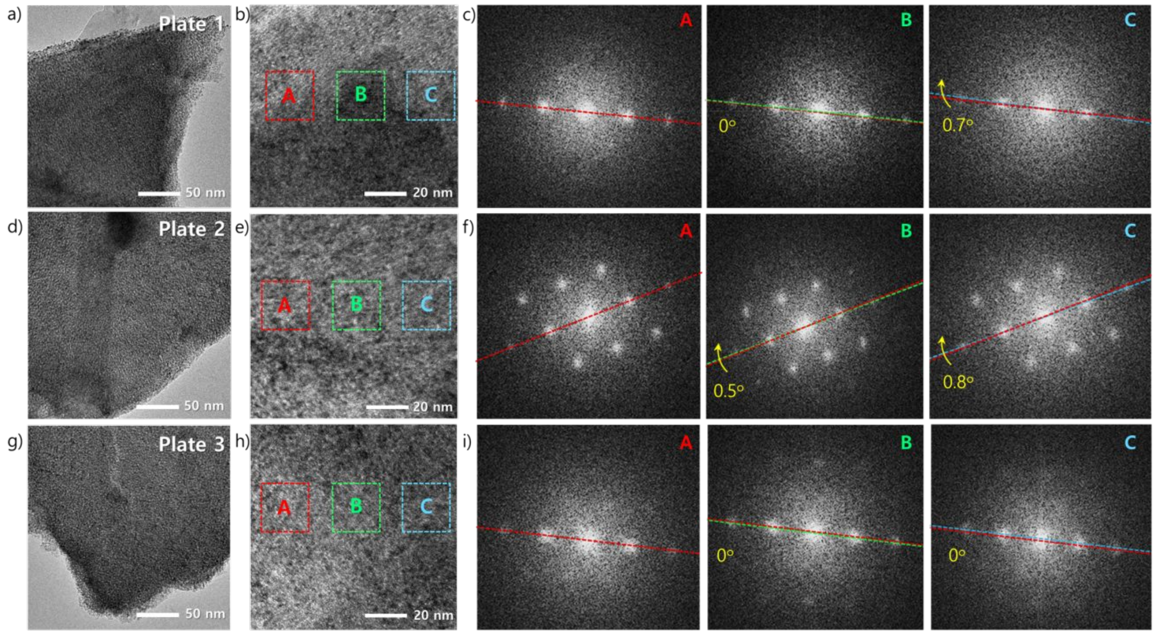

Figure S6. a) TEM image of *D*-Co-NiO nanoplate 1, b) HR-TEM image of nanoplate 1 and c) FFT images of three different positions (A, B, and C) in HR-TEM image of nanoplate 1. d) TEM image of *D*-Co-NiO nanoplate 2, e) HR-TEM image of nanoplate 2 and f) FFT images of three different positions (A, B, and C) in HR-TEM image of nanoplate 2. g) TEM image of *D*-Co-NiO nanoplate 3, h) HR-TEM image of nanoplate 3 and i) FFT images of three different positions (A, B, and C) in nanoplate 3.

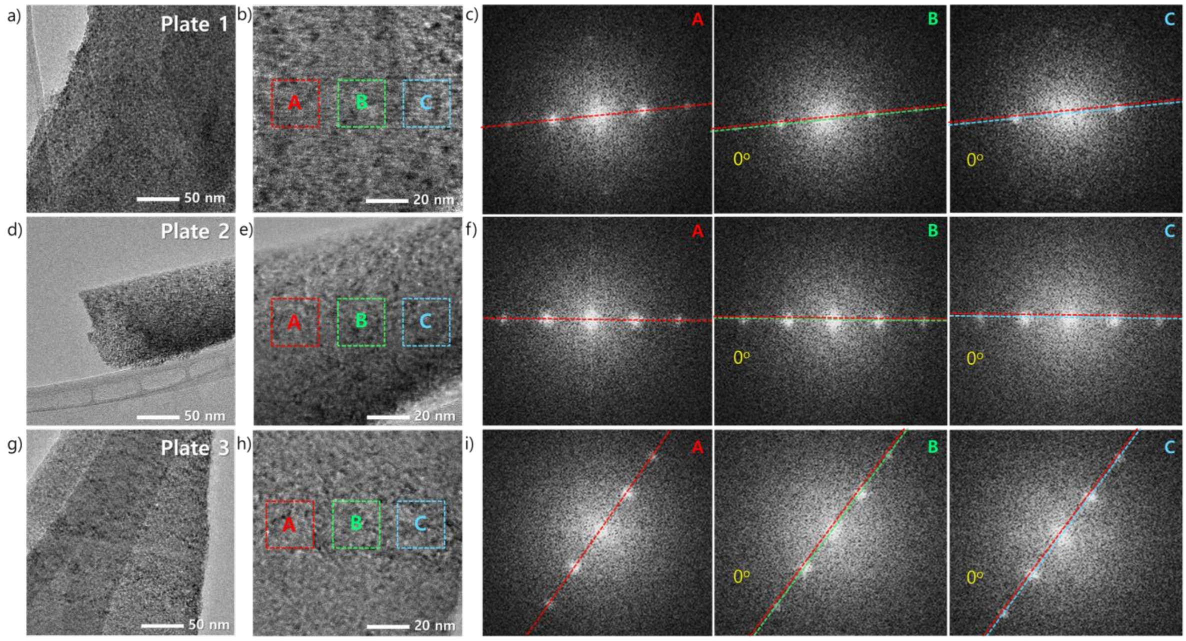

Figure S7. a) TEM image of *DL*-Co-NiO nanoplate 1, b) HR-TEM image of nanoplate 1 and c) FFT images of three different positions (A, B, and C) in HR-TEM image of nanoplate 1. d) TEM image of *DL*-Co-NiO nanoplate 2, e) HR-TEM image of nanoplate 2 and f) FFT images of three different positions (A, B, and C) in HR-TEM image of nanoplate 2. g) TEM image of *DL*-Co-NiO nanoplate 3, h) HR-TEM image of nanoplate 3 and i) FFT images of three different positions (A, B, and C) in nanoplate 3.

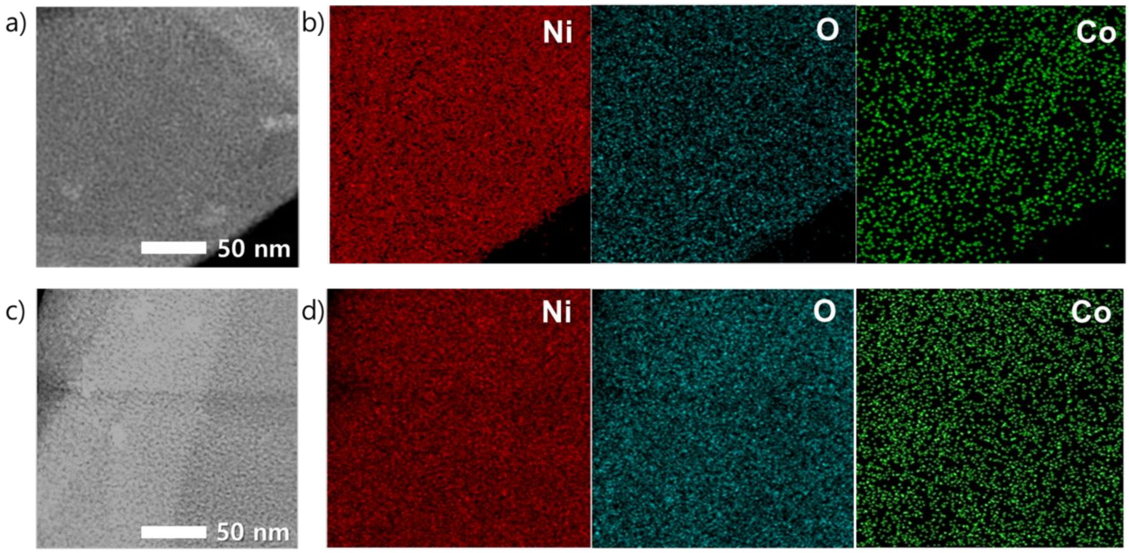

Figure S8. a) STEM image and b) EDX mapping of *D*-Co-NiO nanoplate. c) STEM image and d) EDX mapping of *DL*-Co-NiO nanoplate.

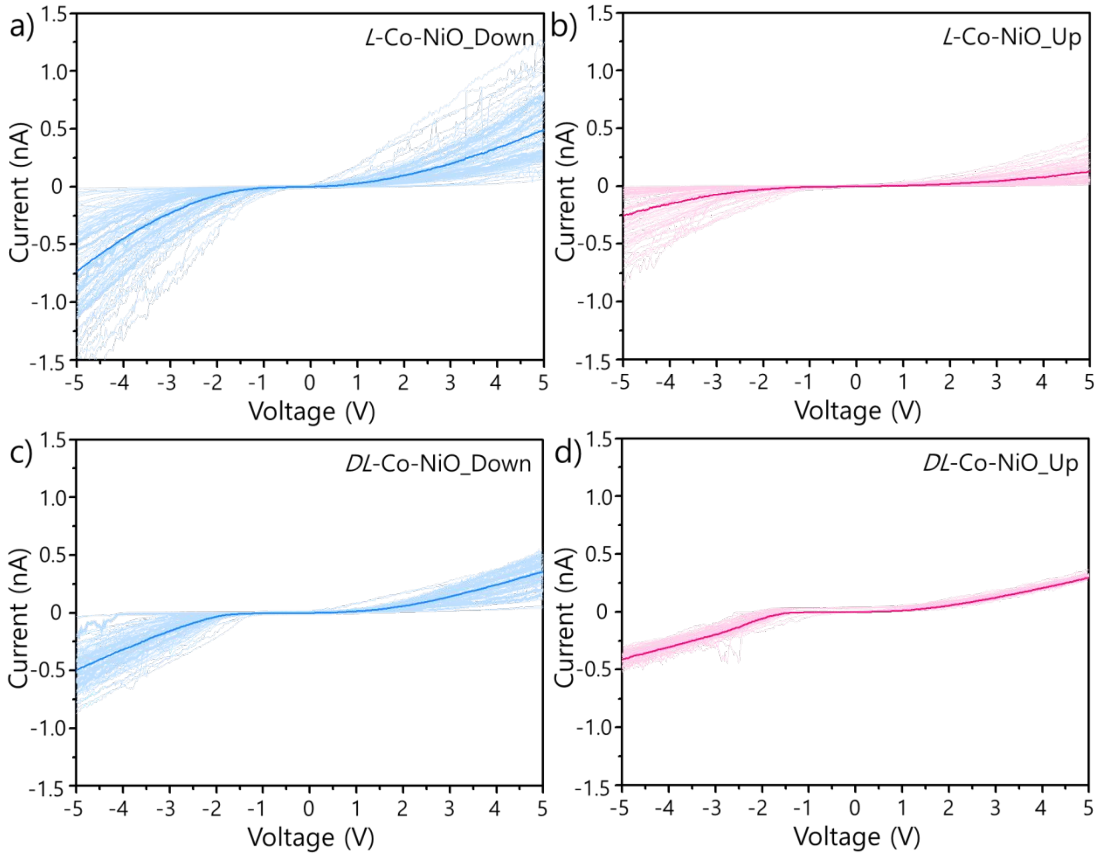

Figure S9. Raw data for *I–V* curves obtained *via* mCP-AFM with a pre-magnetized tip along the upward or downward magnetic field orientation at different positions: a), b) *L*-Co-NiO on FTO and c), d) *DL*-Co-NiO on FTO.

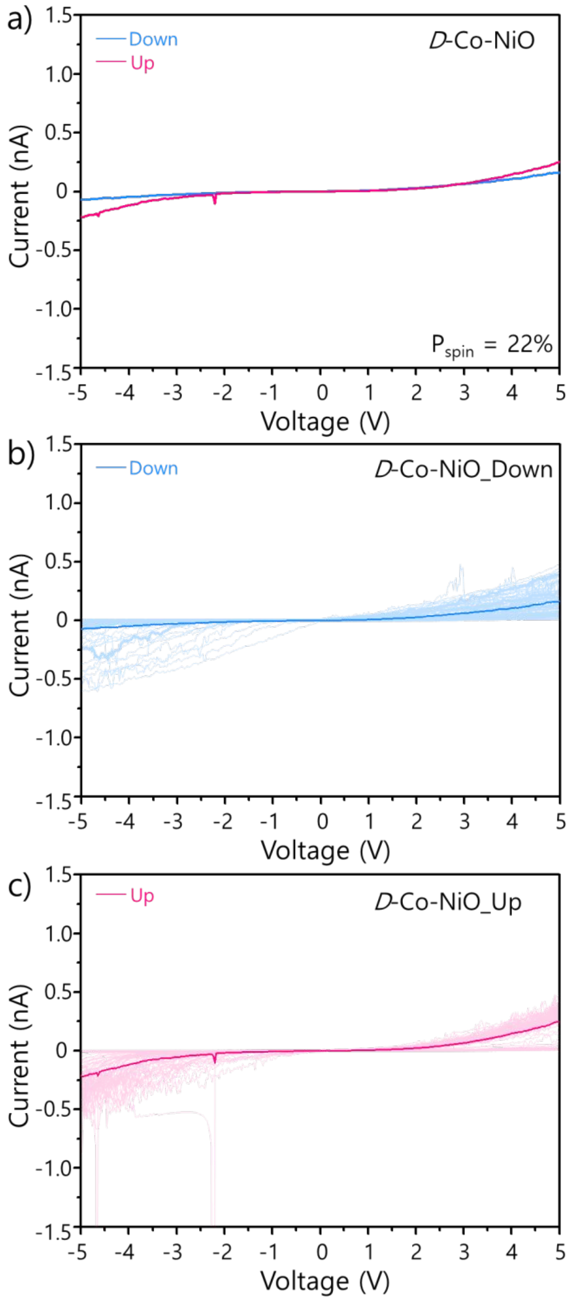

Figure S10. a) Average results and b), c) raw data for *I–V* curves determined *via* mCP-AFM with a pre-magnetized tip along the upward or downward magnetic field orientation at different positions on *D*-Co-NiO on FTO.

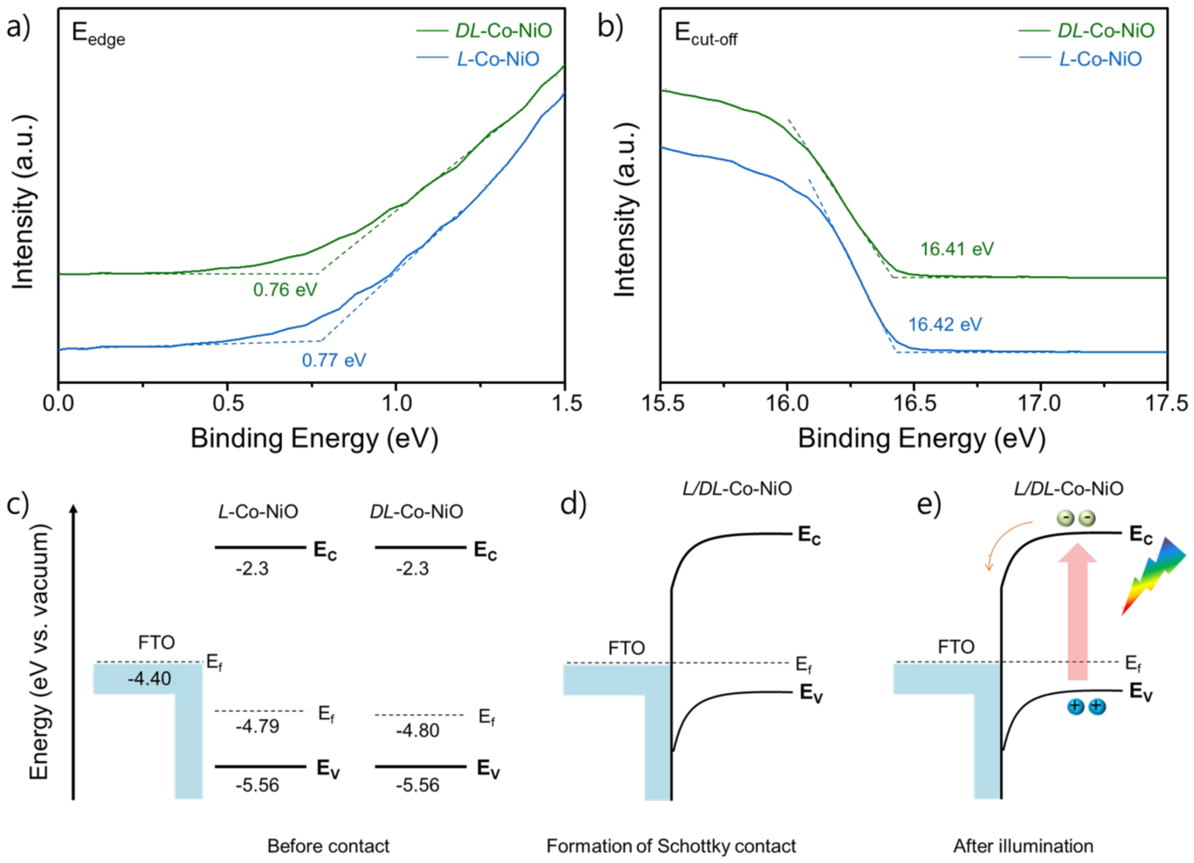

Figure S11. UPS data of a) the valence edges and b) the secondary electron cut-off regions for the *L/DL*-Co-NiO. Scheme for c) band structures of *L/DL*-Co-NiO, d) Schottky contact between *L/DL*-Co-NiO and FTO, and e) transportation of photo-induced electrons between *L/DL*-Co-NiO and FTO.

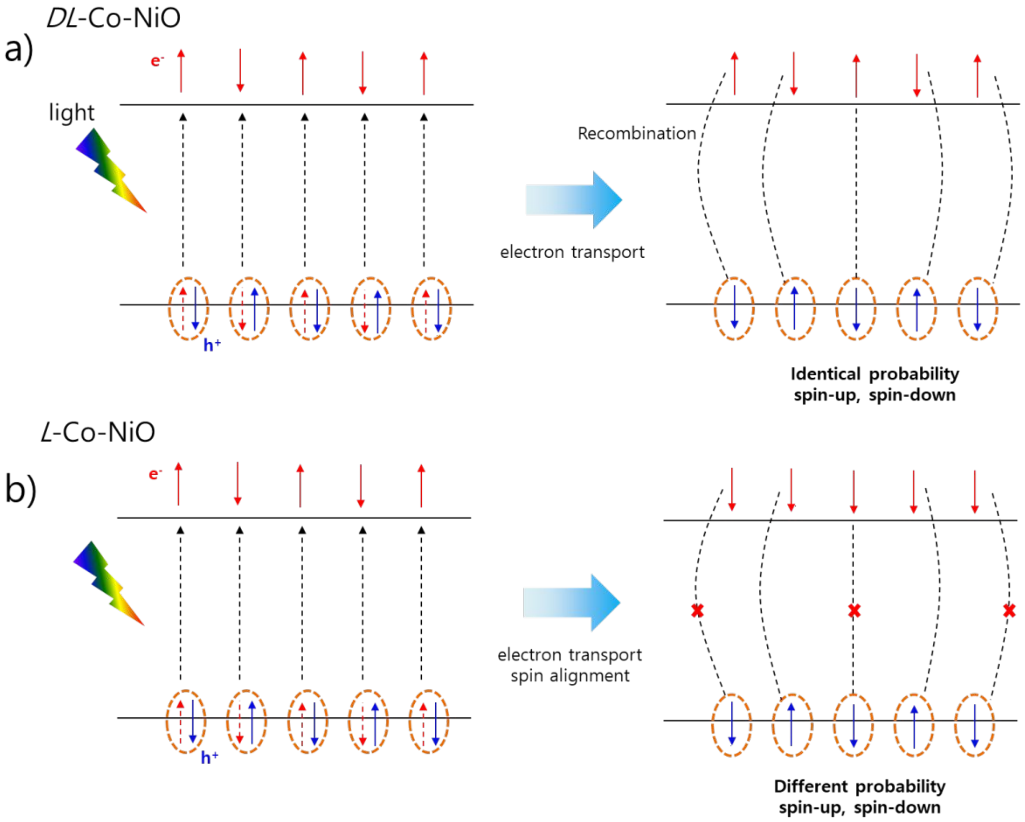

Figure S12. Schematic illustrating the migration of photoinduced charge carriers in a) *DL*-Co-NiO and b) *L*-Co-NiO on FTO during steady-state PL spectroscopy.

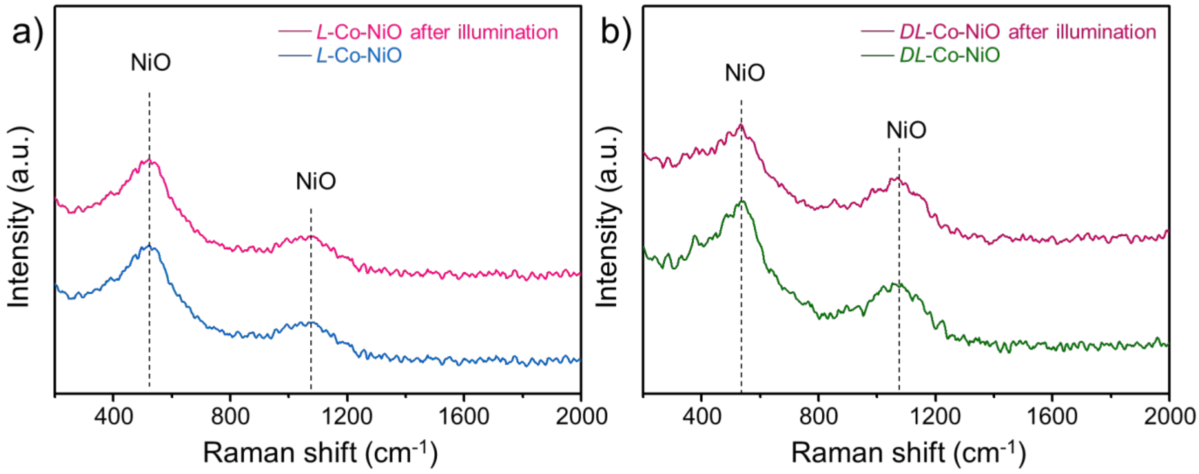

Figure S13. a) Raman spectra for *L*-Co-NiO before and after illumination. b) Raman spectra for *DL*-Co-NiO before and after illumination.

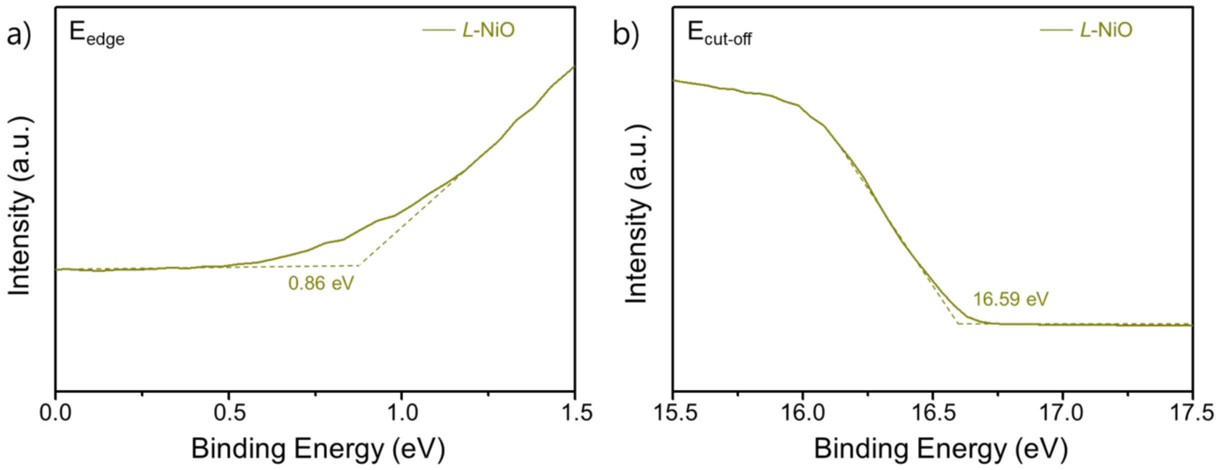

Figure S14. UPS data of a) the valence edge and b) the secondary electron cut-off region for the *L*-NiO.

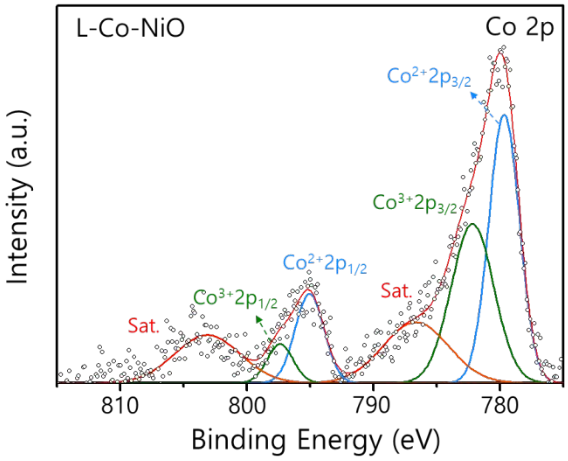

Figure S15. High-resolution XPS spectra of *L*-Co-NiO on NF: deconvolution of Co *2p* peak.

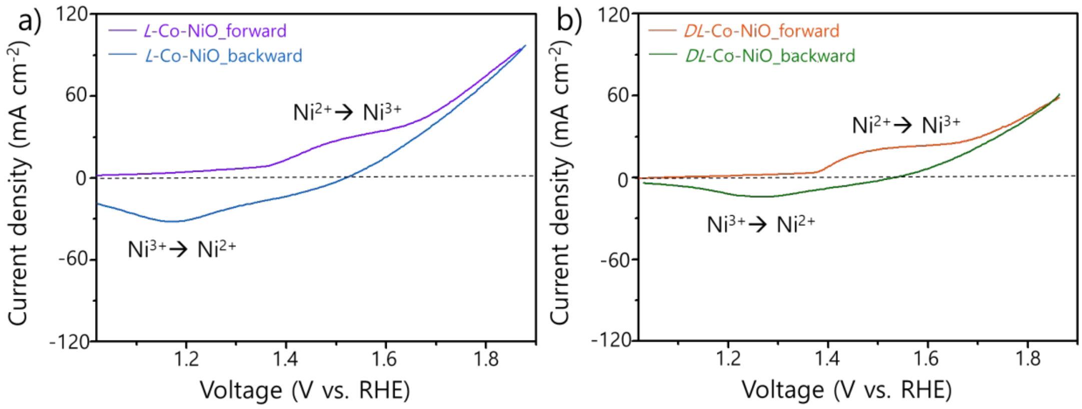

Figure S16. Forward and backward LSV scan curves for a) *L*-Co-NiO and b) *DL*-Co-NiO on NF in 0.2 M KOH electrolyte.

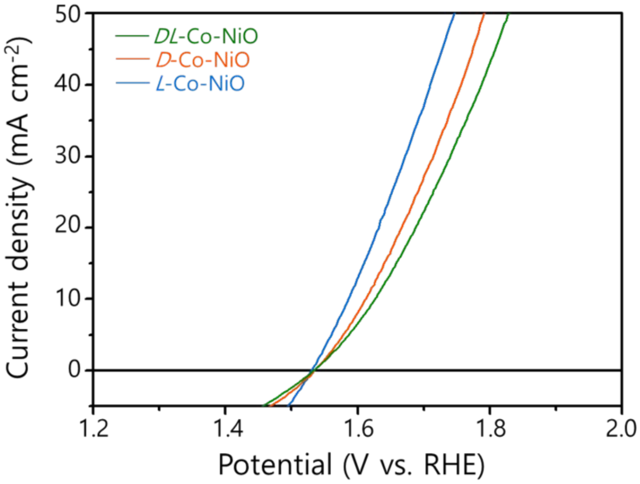

Figure S17. LSV curves for *L*/*D*/*DL*-Co-NiO on NF in 0.2 M KOH electrolyte.

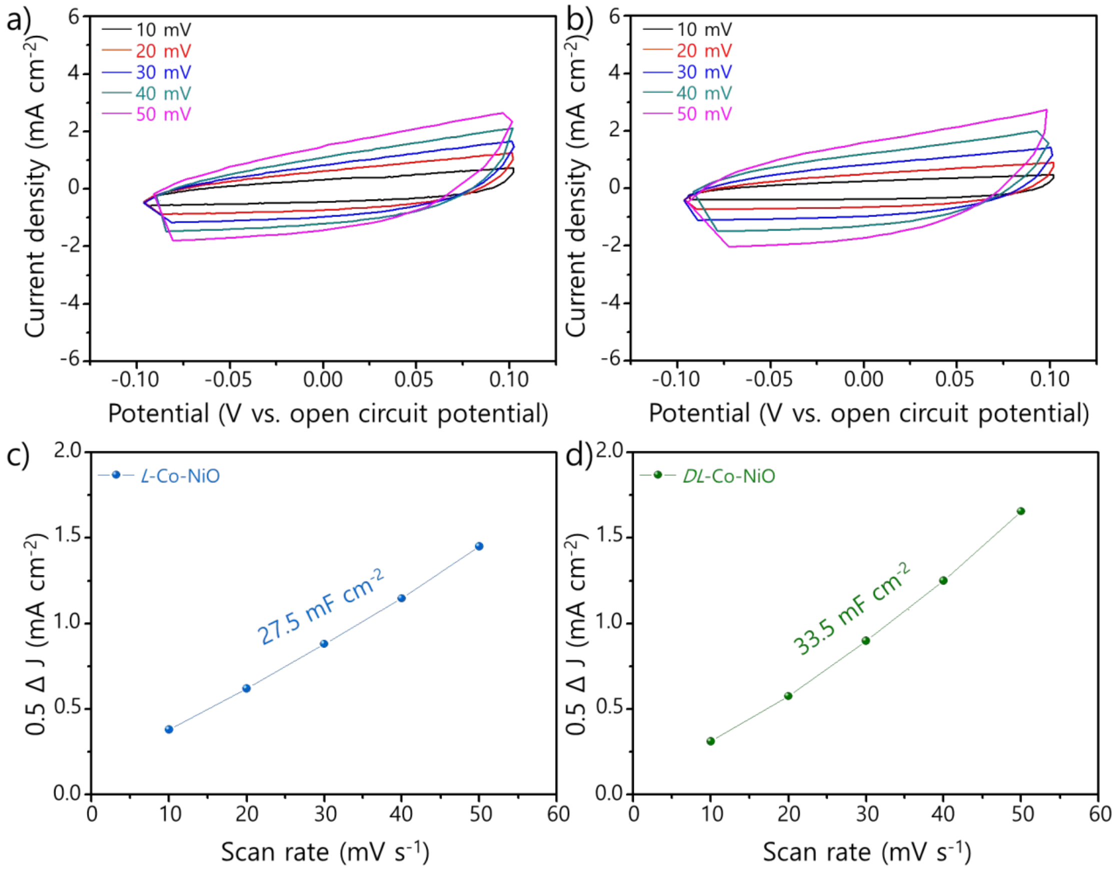

Figure S18. CV curves for a) *L*-Co-NiO and b) *DL*-Co-NiO on NF in 0.2 M KOH electrolyte. Capacitive current densities of c) *L*-Co-NiO and d) *DL*-Co-NiO on NF as a function of scan rate.

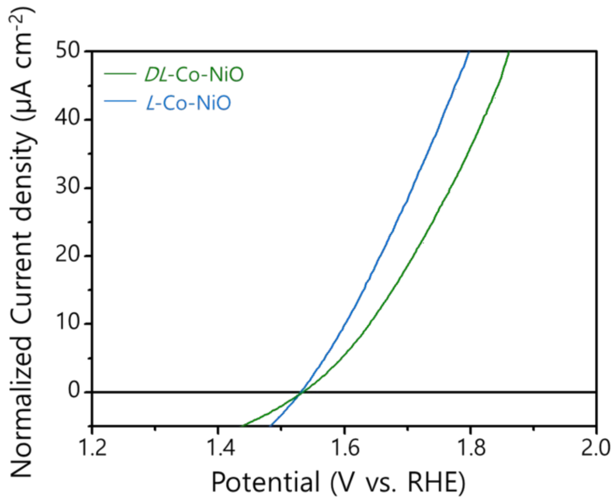

Figure S19. LSV curves for *L*/*DL*-Co-NiO on NF based on ECSA-normalized current density.

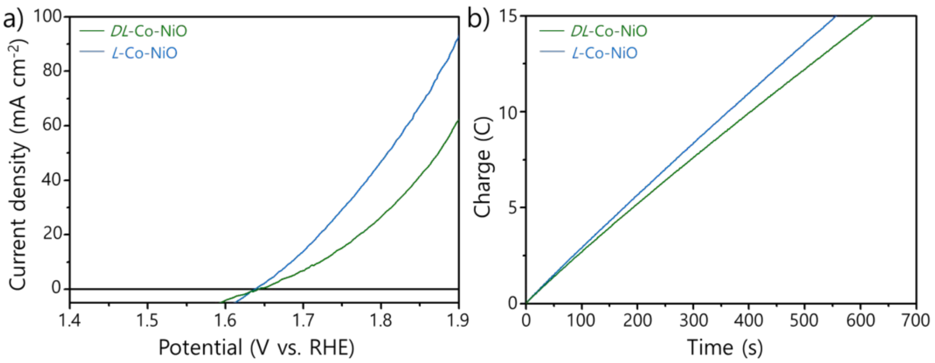

Figure S20. a) LSV curves for *L*/*DL*-Co-NiO on NF in 1 M KPi buffer electrolyte. b) Variation in consumable charge for *L*/*DL*-Co-NiO on NF with time during bulk electrolysis in 1 M KPi buffer electrolyte.

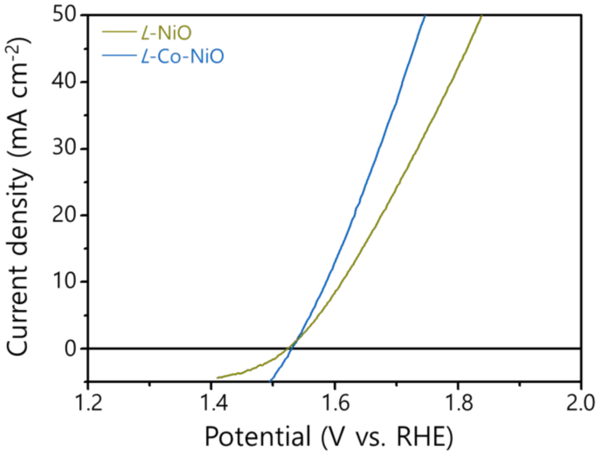

Figure S21. LSV curves for *L*-Co-NiO and *L*-NiO on NF in 0.2 M KOH electrolyte.

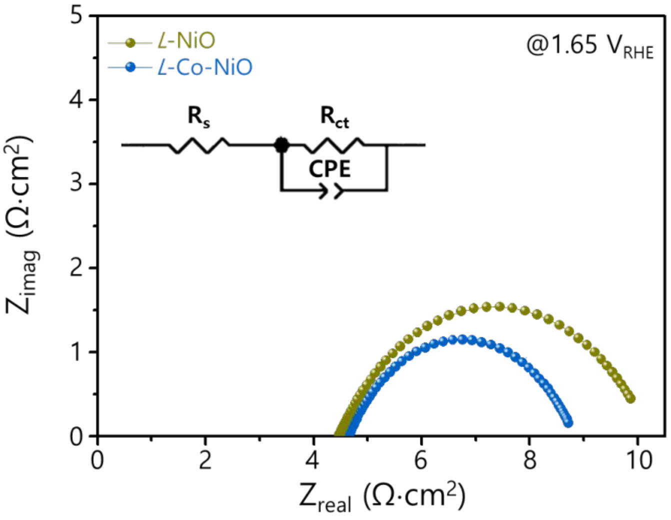

Figure S22. Nyquist plots for *L*-Co-NiO and *L*-NiO on NF in 0.2 M KOH electrolyte.

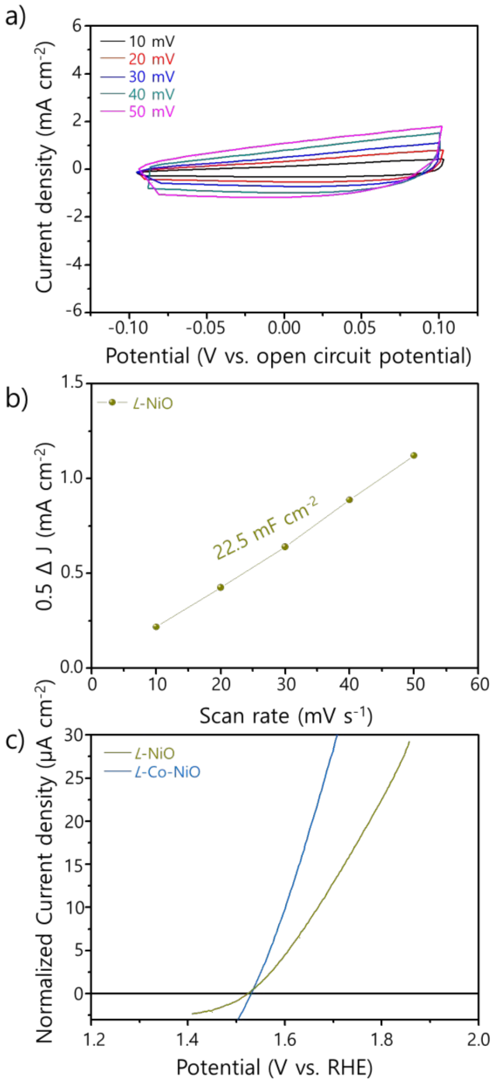

Figure S23. a) CV curves for *L*-NiO on NF in 0.2 M KOH electrolyte. b) Capacitive current densities of *L*-NiO on NF as a function of scan rate. c) LSV curves for *L*-NiO on NF based on ECSA-normalized current density.

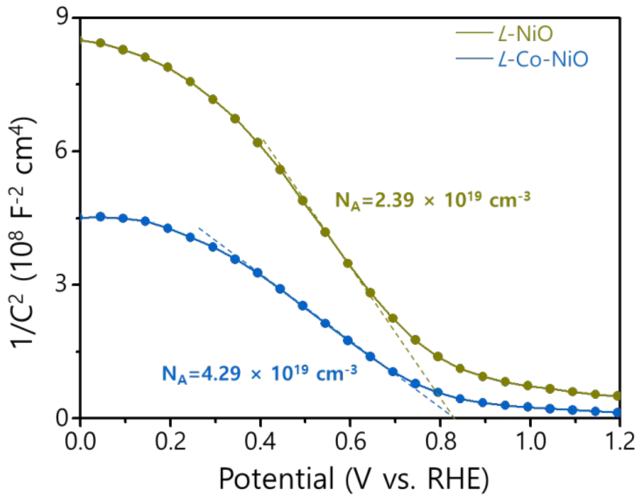

Figure S24. Mott–Schottky plots for *L*-Co-NiO and *L*-NiO on NF in 0.2 M KOH electrolyte.

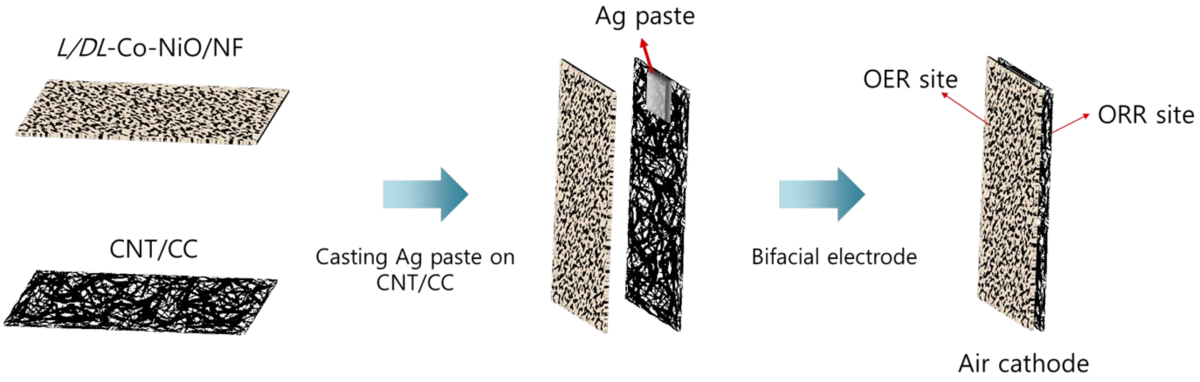

Figure S25. Schematic illustration of *L*/*DL*-Co-NiO@CNT/CC fabrication.

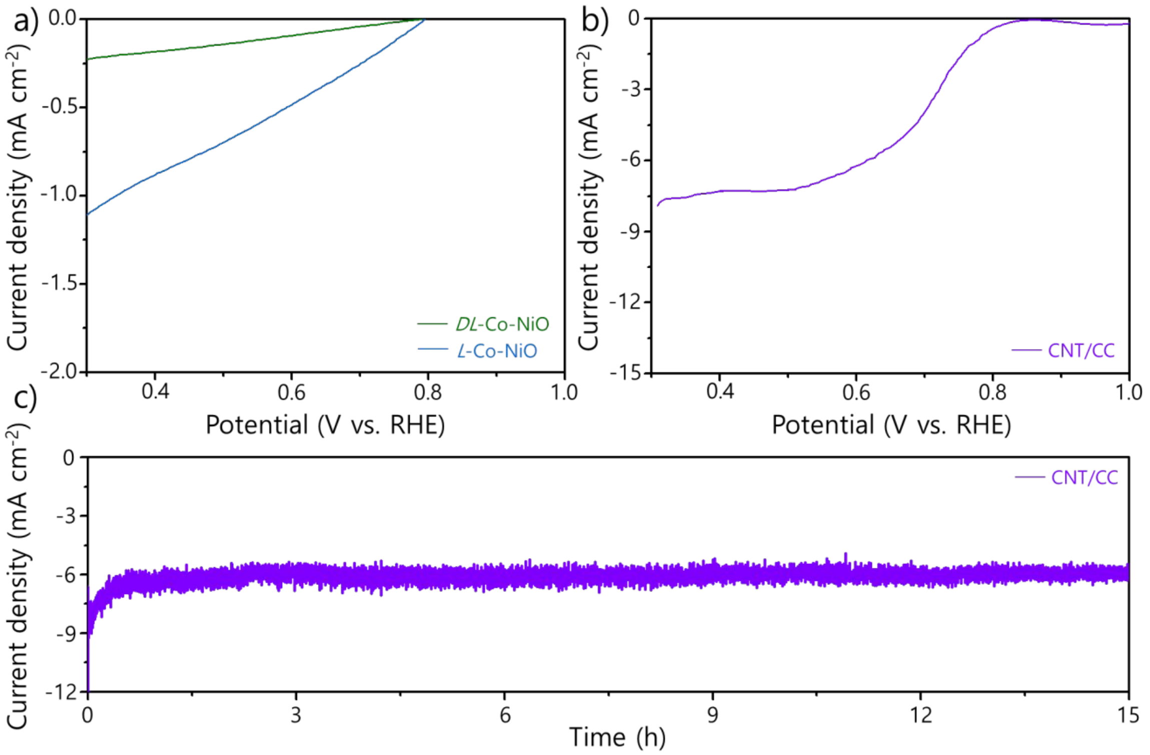

Figure S26. LSV curves for a) *L*/*DL*-Co-NiO on NF and b) CNT/CC toward ORR in O_2-saturated 0.1 M KOH electrolyte. c) Long-term stability of CNT/CC under ORR in O_2-saturated 0.1 M KOH electrolyte.

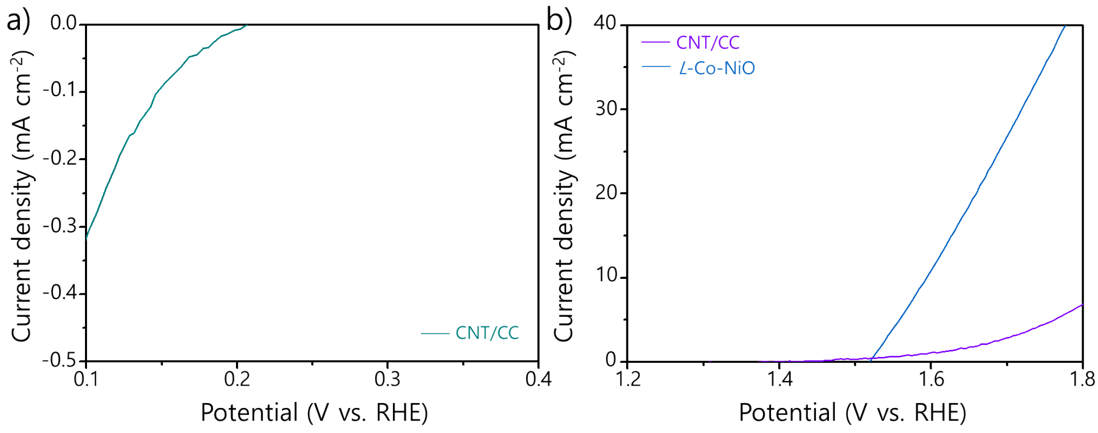

Figure S27. a) LSV curve for CNT/CC in Ar-saturated 0.1 M KOH electrolyte. b) LSV curves for *L*-Co-NiO on NF and CNT/CC toward OER in 0.2 M KOH electrolyte.

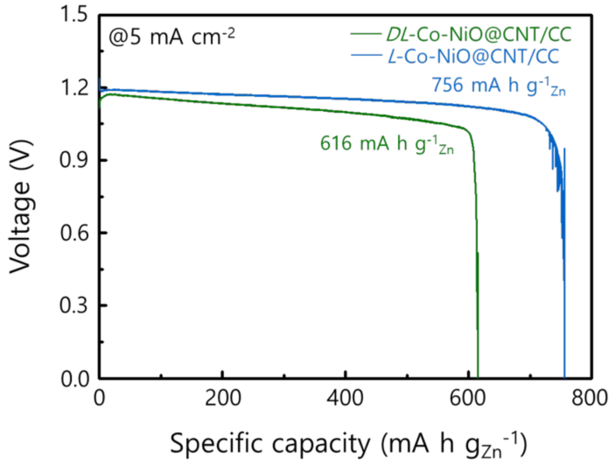

Figure S28. The specific capacities of *L*-Co-NiO@CNT/CC and *DL*-Co-NiO@CNT/CC-based ZAB at 5 mA cm^{-2}.

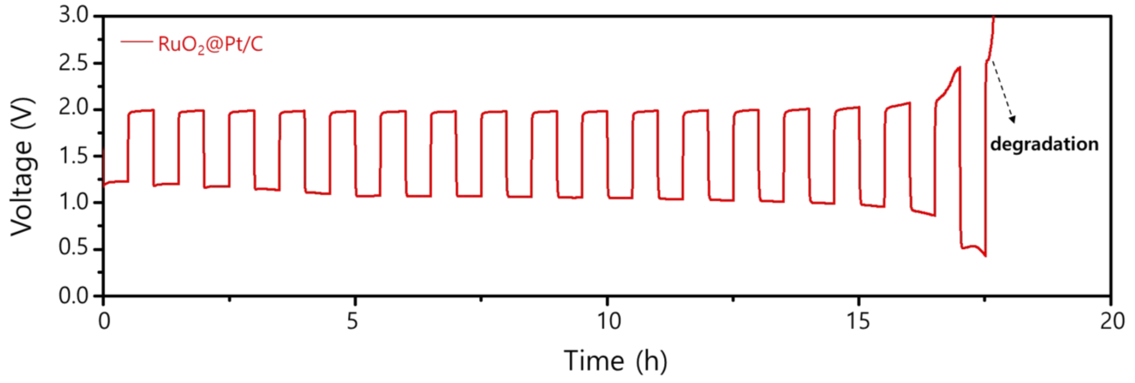

Figure S29. Long-term cycling curve for ZAB with RuO_2@Pt/C.

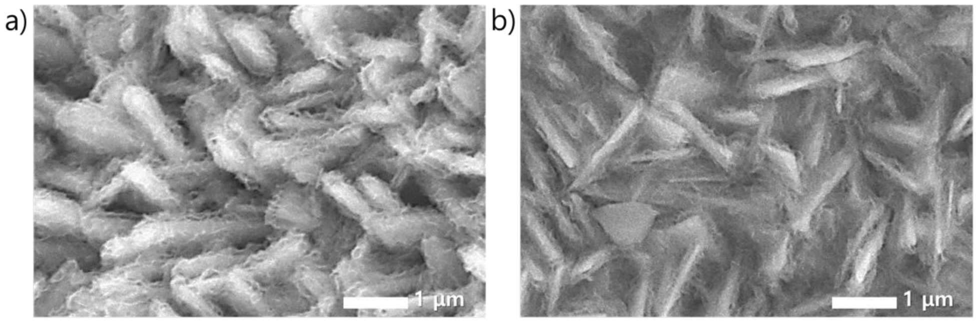

Figure S30. SEM images of a) *L*-Co-NiO on NF and b) *DL*-Co-NiO on NF after cycling.

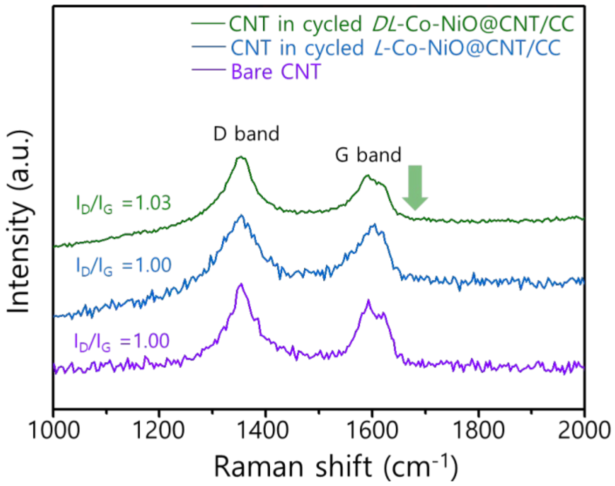

Figure S31. Raman spectra of bare CNT, CNT from cycled *L*-Co-NiO@CNT/CC, and CNT from cycled *DL*-Co-NiO@CNT/CC.

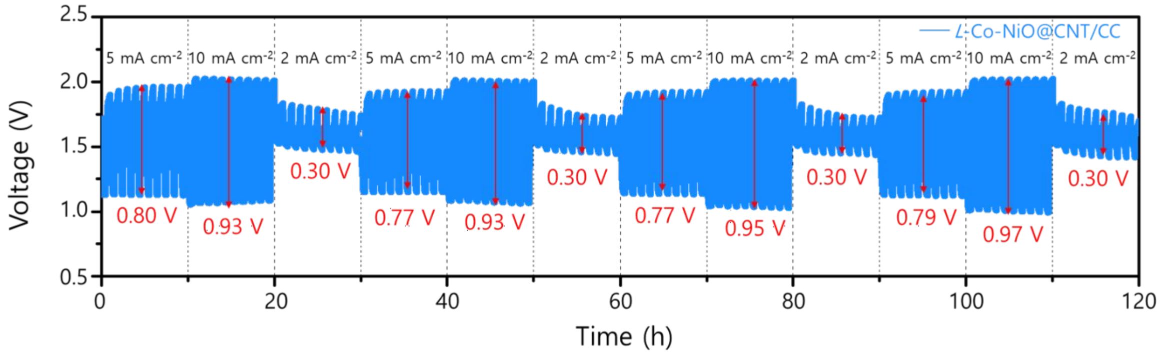

Figure S32. Cycling curve of *L*-Co-NiO@CNT/CC-based ZAB, consecutively operated at 5 mA cm^{-2} for 10 h, 10 mA cm^{-2} for 10 h, and then 2 mA cm^{-2} for 10 h without interruption.

Table S1. Compositional analysis of the *L*-Co-NiO using EDX.

Element	Atomic percent (%)
Ni	44.32
Co	1.84
O	53.84
Total	100

Table S2. Compositional analysis of the *D*-Co-NiO using EDX.

Element	Atomic percent (%)
Ni	39.61
Co	1.43
O	58.96
Total	100

Table S3. Compositional analysis of the *DL*-Co-NiO using EDX.

Element	Atomic percent (%)
Ni	34.59
Co	1.55
O	63.87
Total	100

Table S4. Summarized EIS results for *L*-Co-NiO and *DL*-Co-NiO on NF measured at 1.65 V_{RHE}.

Sample	R_s (Ω·cm^2)	R_{ct} (Ω·cm^2)
L-Co-NiO	4.653	4.175
DL-Co-NiO	4.369	6.005

Table S5. Summarized EIS results for *L*-NiO on NF measured at 1.65 V_{RHE}.

Sample	R_s (Ω·cm^2)	R_{ct} (Ω·cm^2)
L-NiO	4.47	5.753

Table S6. Comparison of durability between the ZAB with *L*-Co-NiO@CNT/CC and recently reported ZABs with low-cost OER catalysts regulated *via* spin modulation.

Air cathode	Initial voltage gap	Final voltage gap	Duration	Decay rate	Ref.
S-doped $LaCoO_3$	0.70 V	0.95 V	100 h	2.5 mV h^{-1}	S1

Ce-doped $LaCoO_3$	0.80 V	1.01 V	160 h	1.3 mV h^{-1}	S2
$La_{0.8}Sr_{0.2}MnO_3$	0.80 V	0.80 V	5 h	0	S3
FeNiPt@C	0.75 V	1.00 V	70 h	3.5 mV h^{-1}	S4
NiOOH/MnFeOOH	1.09 V	1.26 V	350 h	0.46 mV h^{-1}	S5
Fe–N–C	0.84 V	1.13 V	1000 h	0.28 mV h^{-1}	S6
***L*-Co-NiO@CNT/CC**	**0.79 V**	**0.71 V**	**960 h**	**0**	**This study**

REFERENCES

[S1] J. Ran, T. Wang, J. Zhang, Y. Liu, C. Xu, S. Xi, D. Gao, *Chem. Mater.* **2020**, *32*, 3439.

[S2] J. M. Qian, T. T. Wang, Z. M. Zhang, Y. G. Liu, J. F. Li, D. Q. Gao, *Nano Energy* **2020**, *74*, 104948.

[S3] J. M. Qian, H. Zhang, G. Y. Li, L. Jia, X. B. Peng, C. L. Zhong, F. Li, D. L. Chao, D. Q. Gao, *Adv. Funct. Mater.* **2024**, *34*, 2305621.

[S4] Y. D. Pan, Y. W. Li, A. Nairan, U. Khan, Y. Hu, B. X. Wu, L. Sun, L. Zeng, J. K. Gao, *Adv. Sci.* **2024**, *11*, 2308205.

[S5] L. Yang, R. He, M. Botifoll, Y. C. Zhang, Y. Ding, C. Di, C. S. He, Y. Xu, L. Balcells, J. Arbiol, Y. T. Zhou, A. Cabot, *Adv. Mater.* **2024**, *36*, 2400572.

[S6] J. Y. Qiao, C. J. Lu, L. Q. Kong, J. Zhang, Q. Y. Lin, H. B. Huang, C. F. Li, W. He, M. Zhou, Z. M. Sun, *Adv. Funct. Mater.* **2024**, 2409794.
